# Combining and comparing regional SARS-CoV-2 epidemic dynamics in Italy: Bayesian meta-analysis of compartmental models and global sensitivity analysis

**DOI:** 10.3389/fpubh.2022.919456

**Published:** 2022-09-16

**Authors:** Giulia Cereda, Cecilia Viscardi, Michela Baccini

**Affiliations:** ^1^Department of Statistics, Computer Science, Applications, University of Florence, Florence, Italy; ^2^Florence Center for Data Science, University of Florence, Florence, Italy

**Keywords:** global sensitivity analysis (GSA), SARS-CoV-2, infection reproductive number, meta-analysis, meta-regression, cubic regression spline, mean absolute percentage error (MAPE), SIRD compartmental model

## Abstract

During autumn 2020, Italy faced a second important SARS-CoV-2 epidemic wave. We explored the time pattern of the instantaneous reproductive number, *R*_0_(*t*), and estimated the prevalence of infections by region from August to December calibrating SIRD models on COVID-19-related deaths, fixing at values from literature Infection Fatality Rate (IFR) and average infection duration. A Global Sensitivity Analysis (GSA) was performed on the regional SIRD models. Then, we used Bayesian meta-analysis and meta-regression to combine and compare the regional results and investigate their heterogeneity. The meta-analytic *R*_0_(*t*) curves were similar in the Northern and Central regions, while a less peaked curve was estimated for the South. The maximum *R*_0_(*t*) ranged from 2.15 (South) to 2.61 (North) with an increase following school reopening and a decline at the end of October. The predictive performance of the regional models, assessed through cross validation, was good, with a Mean Absolute Percentage Error of 7.2% and 10.9% when considering prediction horizons of 7 and 14 days, respectively. Average temperature, urbanization, characteristics of family medicine and healthcare system, economic dynamism, and use of public transport could partly explain the regional heterogeneity. The GSA indicated the robustness of the regional *R*_0_(*t*) curves to different assumptions on IFR. The infectious period turned out to have a key role in determining the model results, but without compromising between-region comparisons.

## 1. Introduction

After the first SARS-CoV-2 outbreak during spring 2020, Italy faced a stronger second epidemic wave during the autumn of the same year. In order to reduce the rate of contagion and prevent the collapse of the healthcare system, the Italian government introduced regional-level measures of social distancing of different degrees, starting from November 6th 2020. Among others, a national curfew from 10 pm to 5 am was implemented, and the regions were weekly classified as low risk, medium risk, and high risk zones (yellow, orange, and red zones, respectively), according to indicators centrally calculated by the Istituto Superiore di Sanità (ISS). The timing and the degree of the containment measures likely influenced the epidemic dynamics (1), but also socio-economic, demographic characteristics of the population, and environmental factors may have had a role in determining and moderating the level of contagion and its pattern over time. Investigating this issue might be important to prioritizing future interventions and address prevention plans.

Several studies investigated different aspects related to the epidemic dynamics during the first and/or the second wave in Italy by using compartmental models (2–5) or different approaches (6–8). Some studies performed descriptive comparisons among regions (mostly during the first wave) (9–12) or between the first and the second epidemic wave (13). Others explored possible determinants of the heterogeneity in COVID-19 incidence and mortality across the country, focusing on the beginning of the emergency in spring 2020 (14–16).

The aim of our study was to describe the epidemic dynamics in Italy from August 1st 2020 to the end of the same year, in order to obtain an overall picture of the second wave in the country with a special focus on the contagion spread, highlighting and investigating the heterogeneity among regions. We restricted our analysis to the second wave of the COVID-19 epidemic in order to explore associations net of extensive vaccination campaigns. To this end, we adopted a two-step procedure.

First, we estimated a compartmental model of SIRD-type for each region in order to investigate the trend of the contagions over time. Compartmental models, which take their name from the fundamental assumption that at each time during the epidemic the population is divided into homogeneous groups or “compartments”, are widely used in the literature for forecasting and inference purposes, with examples also in COVID-19 research (17). When the interest is to make inference, the model parameters are estimated minimizing the distance between observed data and model predictions (calibration). In our study, we calibrated the regional SIRD models on the daily number of notified COVID-19-related deaths, made publicly available by the Protezione Civile (18). Compared with the number of notified cases, mortality data—although possibly subject to some notification delays—can be considered as more reliable and likely less influenced by the capacity of the healthcare system to detect infections. The calibration procedure allowed us to investigate the behavior of the contagion over time in terms of instantaneous reproductive number *R*_0_(*t*), which quantifies the average number of secondary infections caused by a single infected individual over time, as well as in terms of the number of infected individuals, which can exceed, even by far, the number of the notified ones.

At the second step, we combined and compared the regional results by using Bayesian multivariate and univariate meta-analytic techniques, and we investigated through meta-regressions the possible role of region-level variables in explaining between-region discrepancies in terms of contagion spread.

In the regional SIRD models, we set the infection fatality rate (IFR) and the average time from infection onset to infection resolution to plausible values arising from the literature, in order to assure parameter identifiability. Treating part of the parameters in the compartmental models as fixed is a common practice that poses the problem, usually not addressed, of evaluating the robustness of the results when different values are specified for these parameters (19). In this paper, we performed a sensitivity analysis on the estimated *R*_0_(*t*) curve and prevalence of infections, by changing one at a time the values of IFR and infection duration in the SIRD models. Additionally, we implemented a Global Sensitivity Analysis (GSA) procedure (20) to quantify and characterize the uncertainty around the calibration results that propagated from the uncertainty around the values of those parameters that were not the object of the inference. Despite GSA is not yet widely used in epidemiology, it appears as one of the recommendations in the Manifesto by Saltelli et al. (21), which offers a critical view of modeling in time of pandemic.

Finally, as an ancillary result, we obtained an evaluation of the submerged fraction of contagion and an indirect assessment of the admissible IFR values, by comparing the number of infections estimated by the regional SIRD models, which by definition includes both notified and non-notified cases, with the observed number of notified infections reported by the Protezione Civile (18).

## 2. Materials and methods

### 2.1. Data

For our analyses, we used the national database on the evolution of the COVID-19 pandemic, made available on a daily basis by the Protezione Civile (18). This database collects the number of notified infections, COVID-19-related hospitalizations and deaths and recovered subjects by region. In the estimation phase, we used the daily number of COVID-19-related deaths from August 1st 2020 to January 14th 2021 for all Italian regions, and the number of notified infections circulating on July 31st 2020. The daily number of new infections and the daily number of circulating infections from August 1st 2020 to January 14th 2021 were used in a descriptive way to indirectly assess the submerged fraction of contagion. We extended the study period until January 14th to obtain a more stable inference on the month of December, taking into account that deaths observed today may result from contagions that occurred weeks ago.

We merged data of the two autonomous provinces of Trentino Alto Adige (Bolzano-Alto Adige and Trento) that were provided separately. We removed from the death counts of Emilia-Romagna 154 cases that, although happened during spring 2020, have been added to the data set on August 15th. In the map of Supplementary Figure S1, we represented, for each Italian region, the population size and the total number of COVID-19-related deaths notified during the study period (see also Supplementary Table S1).

We collected socio-demographic and economic indicators measured at the regional level for 2020 or for the last available year from the website of the Italian National Institute of Statistics (ISTAT) (22) (see Section 2.4 for further details). We calculated the regional average temperatures during the study period from the daily temperature measurements reported on the website www.ilmeteo.it.

### 2.2. Regional SIRD models

For each region, we adopted a compartmental model of SIRD type, described by the following system of differential equations:


(1) $$\begin{array}{l} \{\begin{array}{l} \frac{dS(t)}{dt}=-\beta (t)\frac{S(t-1)}{S(0)}I(t-1)\\ \frac{dI(t)}{dt}=\beta (t)\frac{S(t-1)}{S(0)}I(t-1)-\alpha I(t-1)-\delta I(t-1)\\ \frac{dR(t)}{dt}=\alpha I(t-1)\\ \frac{dD(t)}{dt}=\delta I(t-1) \end{array} \end{array}$$

where *S*(*t*), *I*(*t*), *R*(*t*) and *D*(*t*) are the sizes of the Susceptible, Infected, Recovered and Deceased compartments at time *t* (23). For each region, we fixed *I*(0) to the number of notified infections circulating at time 0 (July 31st) as reported by the Protezione Civile (18), from now on denoted by *i*_0_. Denoting by *N* the regional population size as of January 1st 2020 (22), we approximated the number of susceptible people at time 0 as *S*(0) = *N*−*i*_0_, assuming that the total number of individuals who had become immunized since the start of the pandemic was negligible with respect to *N* (see Supplementary Table S1). We set *D*(0) = 0 and *R*(0) = 0, thus starting to count deaths and recoveries from August 1st. The parameters α and δ are the transition rates from the compartment of the infected individuals to the compartments of the recovered and deceased ones, respectively. They depend on the IFR, denoted by *p*, and on the average times from infection to death and from infection to recovery, denoted by *T*_*D*_ and *T*_*R*_, respectively. Having set *T*_*D*_ = *T*_*R*_ = *T*, the following relationships hold: $\alpha =\frac{1-p}{T}$, $\delta =\frac{p}{T}$ (24). The infection rate β(*t*) is related to the instantaneous reproductive number *R*_0_(*t*), modeled as time-dependent, as follows:


(2) $$\begin{array}{r} \beta \left(t\right)=R_{0}\left(t\right)\left(\alpha +\delta \right)=\frac{R_{0}\left(t\right)}{T}. \end{array}$$

At the beginning of the epidemic, *R*_0_(*t*) corresponds to the basic reproductive number, defined as the number of secondary infections generated by the first infected individual in the population. *R*_0_(*t*) is also related to the effective reproductive number, *R*_*t*_ = *R*_0_(*t*)*S*(*t*)/*S*(0) (25), that measures the actual transmission at a specific time accounting for the natural depletion of susceptible individuals as the contagion spreads. *R*_*t*_ departs from *R*_0_(*t*) only if the level of immunity in the population is not negligible, with a ratio *S*(*t*)/*S*(0) far from 1.

To get a flexible estimate of *R*_0_(*t*), we modeled it through a natural cubic regression spline (26), with 4 internal equi-spaced knots (6 degrees of freedom): *R*_0_(*t*) = *s*(*t*; ***ϑ***), where ***ϑ*** is a vector of unknown coefficients, to be estimated.

We assured parameter identifiability by fixing in the model *T* = 14 days and *p* = 1.15%. The value *p* = 1.15% is the IFR estimate reported for the upper-income countries by the Imperial College COVID-19 response team (27). It is also consistent with the value of 1.14% estimated for Italy by the Italian Institute for International Political Studies (28) and used in a previous paper by the authors (29). Regarding *T*, the value of 14 days is in line with both the median time from symptoms onset to death reported by ISS for Italy (12 days) (30) and the estimated average time from infection onset to recovery of 13.4 days arisen from a meta-analysis (31). We explored these choices on *p* and *T* through one-factor-at-a-time sensitivity analysis and GSA.

In the estimation phase, we discretized the differential equations in (1) and evaluated the size of the compartments by considering unit time intervals (details in Supplementary Section S1). This allowed us to estimate the model minimizing over ***ϑ*** the following sum of squares:


(3) $$\begin{array}{c} Q(\vartheta )=\sum_{t=1}^{K}D(t;\vartheta )-D^{\text{obs}}{\left(t\right)}^{2}, \end{array}$$

where *t* = 1 corresponds to August 1st 2020, *t* = *K* to January 14th 2021, and *D*^obs^(*t*) denotes the cumulative number of deaths observed from August 1st 2020.

We performed the minimization of (3) through the *auglag* function of the nloptr package of R software (http://ab-initio.mit.edu/nlopt), constraining the function *R*_0_(*t*) to positive values. We ran the estimation algorithm 100 times, using different initial values sampled from a multivariate grid defined on the values of ***ϑ***. Among the 100 parameters estimates thus obtained, we selected the estimate $\hat{\vartheta }$ associated to the lowest value of *Q*(·).

We implemented a parametric bootstrap procedure, in order to quantify the sampling variability around the estimates. Following a consolidated procedure (32, 33), we assumed a Negative Binomial distribution on the daily increments of the estimated time series $D\left(t;\hat{\vartheta }\right)$ and generated 500 bootstrap samples to be used as observed time series in as many calibrations. The 90% confidence intervals or bands for the quantities of interest have been calculated as the 5th and 95th percentiles of the bootstrap distributions (see Supplementary Section S2 for further details).

We adopted a cross-validation approach similar to the one proposed in Šušteršič et al. (34) to assess the performance of our model in predicting COVID-19-related deaths in terms of Mean Absolute Percentage Error (MAPE) (10). We focused on prediction horizons of 7 and 14 days. Details on the validation procedure are reported in Supplementary Section S3.

### 2.3. One-factor-at-a-time sensitivity analysis and GSA

To investigate how changes in the values of *p* and *T* affected the estimates of *R*_0_(*t*) and the shapes of the epidemiological curves arising from the regional SIRD models, we repeated the analyses for *p* = 0.78%, 1.79% and *T* = 10, 18. The values *p* = 0.78% and *p* = 1.79% are the 95% confidence interval bounds of the IFR estimate in Brazeau et al. (27). We performed an additional analysis fixing *p* = 0.5%, as an extreme lower bound for the IFR. The value *T* = 10 is consistent with the 95% lower bound of the estimated mean time of Byrne et al. (31), while *T* = 18 is the estimated mean duration of the maximal infectious period from the same study.

Then, we went beyond the previous one-factor-at-a-time sensitivity analysis by performing a GSA, calculating the Sobol's variance indexes (20). Given a function that relates inputs to outputs, the GSA explores how the outputs vary as the inputs change, to determine the inputs most contributing to the behavior of the outputs (factor prioritization), finding non-influential inputs (model simplification), and investigating interaction effects between inputs. This can be done by propagating the uncertainty around the inputs to the outputs *via* MC simulations, then using the Sobol's decomposition of the variance of each output thus obtained, and apportioning it among the different inputs (35) (see Supplementary Section S4). The contribution of each input to the output variance can be quantified by computing first order indexes (and superior order indexes) and total variance indexes. In particular, the first order index of a given input represents the proportion of the output variance which is due to the main effect of the input (i.e., the first-order effect), while the total effect index represents the proportion of the output variance which is due to the main effect of the input and all its interactions with the other inputs (higher-order effects) (see Supplementary Section S4).

In our application, we considered as inputs the fixed parameters of the SIRD model (*p*, *T*_*R*_, *T*_*D*_, *I*(0)) and as outputs the parameters estimated by calibration, as well as derived quantities, such as the maximum and minimum *R*_0_(*t*), the peak of infections, and the dates at which they occurred, together with the first date when *R*_0_(*t*) reached the threshold of 1 after the maximum infection peak. The model was the calibration algorithm, given the observed data.

We calculated the first order and total variance indexes of each input on each output, relying on the results of 5'040 MC simulations, that in our case corresponded to 5'040 calibrations of the SIRD model. Each calibration was performed under a different combination of inputs, obtained by sampling *p* from the continuous uniform distribution U[0.0078,0.0179] (27), and the transition times and *I*(0) from the following discrete uniform distributions: $T_{R}\sim U\left\{7,21\right\}$, $T_{D}\sim U\left\{7,21\right\}$, $I\left(0\right)\sim U\left\{i_{0},3i_{0}\right\}$. For each input, the aggregate total variance index on the vector ***ϑ*** was calculated as a weighted average of the total variance indexes of the single spline coefficients, with weights proportional to the output variability (36).

Given the huge computational effort required by the GSA implementation, we performed it on a virtual machine with 16 vCPU only for one region (Tuscany). However, we expect that the results can be generalized to the other regions. The GSA was conducted by using the *soboljansen* and the *sobolMultOut* functions of the R package sensitivity (https://cran.r-project.org/web/packages/sensitivity/sensitivity.pdf).

### 2.4. Bayesian meta-analyses and meta-regressions

We used a Bayesian multivariate random effects meta-analysis model to combine the estimated region-specific *R*_0_(*t*) curves, accounting for the heterogeneity among regions, and to combine the regional estimates of the monthly average prevalence of infection from September to December.

Additionally, we conducted Bayesian univariate random effects meta-analyses on the following quantities obtained from the regional *R*_0_(*t*) curves:

- average value of *R*_0_(*t*) from October 1st to December 31st;
- maximum value of *R*_0_(*t*) during the study period;
- variation of *R*_0_(*t*) during the 4 weeks following November 6th, date of the introduction of social distancing measures by the central government, with a classification of the regions according to three risk levels;
- variation of *R*_0_(*t*) during the 4 weeks following the beginning of the school.

For the variation of *R*_0_(*t*) during the 4 weeks following November 6th, we performed also meta-analyses by risk level assigned to the region in the first week of the introduction of restrictions. Univariate and multivariate meta-analyses were conducted on all regions and separately by geographical area: Southern regions (Basilicata, Calabria, Campania, Molise, Puglia, Sardegna, Sicilia), Central regions (Abruzzo, Lazio, Marche, Toscana, Umbria), Northern regions (Emilia Romagna, Friuli Venezia Giulia, Liguria, Lombardia, Piemonte, Trentino, Valle d'Aosta, Veneto).

Finally, Bayesian meta-regression analyses were performed on the average *R*_0_(*t*) from October 1st to December 31st in order to investigate possible sources of between-region heterogeneity. Specifically, we evaluated as possible effect modifiers the following variables measured at the regional level (for 2020 or for the last available year): percentage of people with at least two chronic diseases in the population, number of general practitioners per 10'000 residents, number of pediatricians per 10'000 children, number of hospitals per 1,000 residents, percentage of public hospitals over the total number of hospitals, aging index (number of over 65 per 100 individuals younger than 15), mean age of the population, average size of households, percentage of people aged 24–65 with an academic degree per 10'000 residents, schooling index (percentage of the individuals aged 20–24 who have at least a high school diploma), percentage of children attending kindergartens, percentage of workers using public transport to go to work, percentage of people aged 0–34 using public transport to go to school, poverty index (percentage of people living in households below the poverty threshold), employment rate (percentage of employed persons in the class of age 15–64), tourism rate (days of presence of tourists during the year per inhabitant), total energy consumption of industries and manufactures, percentage of residents living in high-urbanization areas, average temperature from October to December 2020 (see Supplementary Table S2 for details on meta-regressors and related references). We specified separate meta-regression models each of which included only one meta-regressor.

In all analyses, non-informative priors were assumed on the model hyperparameters. We get a sample from the joint posterior distribution of the hyperparameters *via* MCMC algorithm. A description of models and software used for the analysis is reported in Supplementary Section S5.

## 3. Results

### 3.1. Results of the main analysis (p = 1.15%, T = 14)

The fit of the SIRD model was good for all regions, with the expected cumulative deaths close to the observed ones (Supplementary Figure S2).

**Figure 1:**
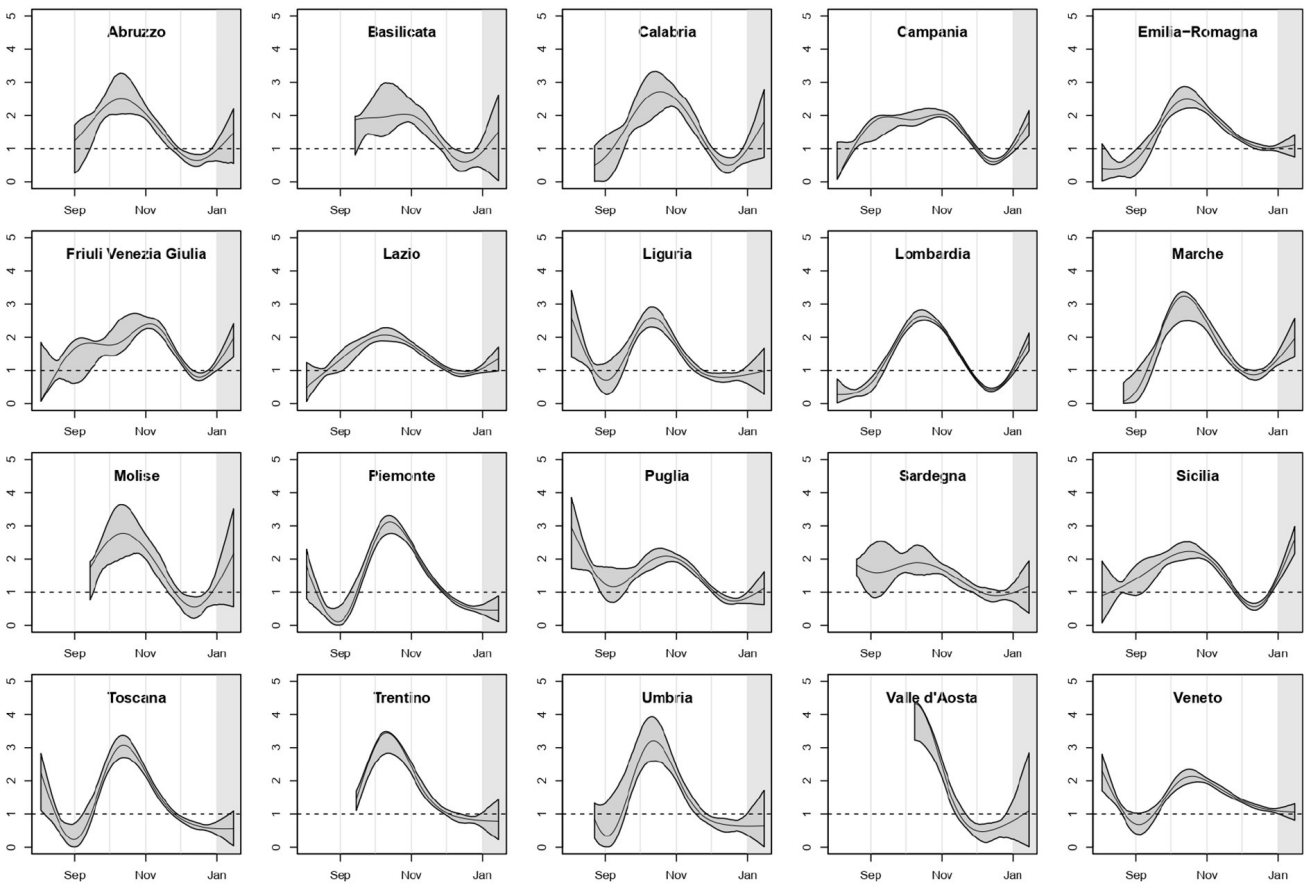
Estimated *R*_0_(*t*) with point-wise 90% confidence bands, by region; *p* = 1.15%, *T* = 14 days.

In Figure 1 we reported the estimated curves of *R*_0_(*t*) arising from the regional SIRD models with their 90% point-wise confidence bands. For the regions where the first COVID-19-related death was observed after the second week of August, the curve was shown starting 14 days before the first death, because of the extremely poor information on the previous period. We highlighted in gray the last 14 days of the study period, included in the analysis to make estimates more stable for the last weeks of 2020.

The pattern of *R*_0_(*t*) was heterogeneous among regions, but a mid/late-October peak was visible as the culmination of a growth started in early/mid-September, with only two exceptions: Campania and Friuli Venezia Giulia. In several regions, *R*_0_(*t*) declined and then increased again starting from mid-December.

**Figure 2:**
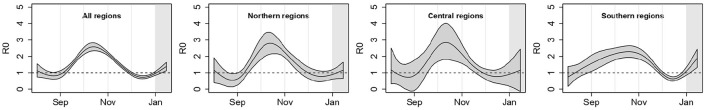
Posterior mean of the meta-analytic curves of *R*_0_(*t*) with point-wise 90% credible bands for the entire country and by geographical area; *p* = 1.15%, *T* = 14 days.

Figure 2 summarizes these regional results showing the overall meta-analytic Italian *R*_0_(*t*) curve and the meta-analytic curves by geographical area, obtained from Bayesian multivariate meta-analyses (posterior mean curve and 90% point-wise credible intervals). It is quite evident that during the second epidemic wave the shape of *R*_0_(*t*) was similar in the Northern and Central regions, with an initial increase and a clear peak around the middle of October. On the contrary, the overall curve for Southern regions was flatter, without the initial decrease. The combination of all the regional curves leads to an overall *R*_0_(*t*) curve (left plot) similar to the one obtained for the Northern and Central regions, even though with tighter credible bands.

**Figure 3:**
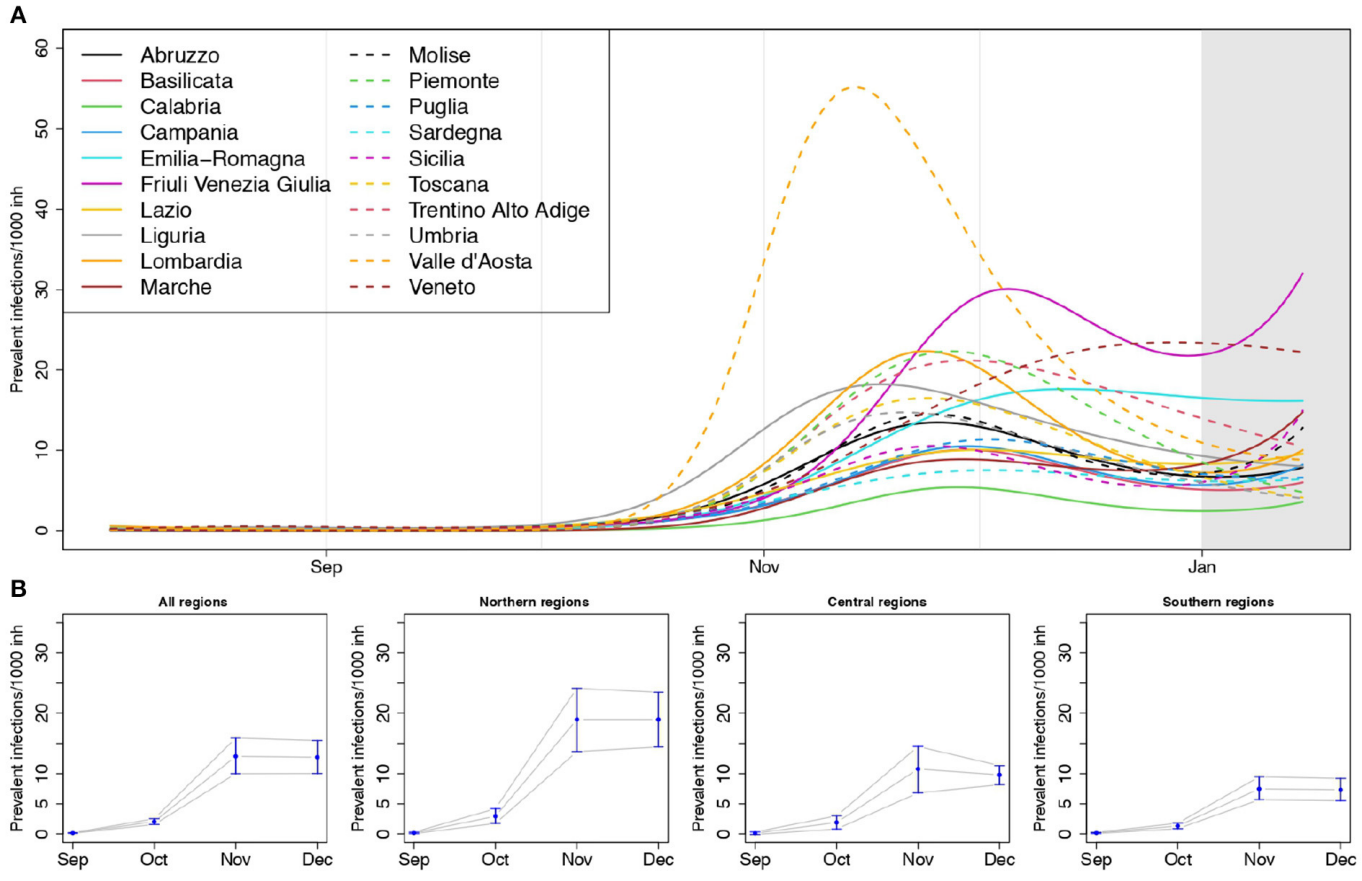
**(A)** Estimated prevalence of infections (number of circulating infections over 1,000 inhabitants) by region, and **(B)** posterior mean of the meta-analytic monthly-average prevalences, for the entire country and by geographical areas, with 90% credible intervals (lower panel); *p* = 1.15%, *T* = 14 days.

In Figure 3A, we reported the regional curves describing the prevalence of infections per 1, 000 inhabitants over time, arising from the SIRD models (see also Supplementary Figures S3, S4 and Supplementary Table S3). These estimates are inclusive of detected and undetected cases, thus, in principle, they should be an upper bound for the number of notified cases provided by the Protezione Civile (see Section 3.3 for a discussion about this point). Valle d'Aosta exhibited the largest peak of prevalence, reached in the first half of November, with more than 50 circulating infections every 1,000 inhabitants. It was followed—even though with less than half the value of its prevalence—by Friuli Venezia Giulia, Veneto, Piemonte, Lombardia, Trentino Alto Adige and then by Liguria and Emilia Romagna. The lowest prevalence was estimated for Calabria. Liguria and Valle d'Aosta reached the peak of circulating infections before the other regions, while Veneto was the last one.

The overall estimates of the average monthly prevalence arising from the Bayesian multivariate meta-analysis (Figure 3B) highlight how the prevalence of infections in the Northern regions was larger than in the Central and Southern ones, exceeding on average 15 cases every 1,000 inhabitants during the month of November.

The values of the MAPE averaged over the regions were 7.2% and 10.9% for the 7 days and 14 days prediction horizons, respectively. The region-specific MAPEs at 7 days ranged from 3.3% (Veneto) to 22.6% (Valle d'Aosta), those at 14 days ranged from 4.1% (Basilicata) to 26.0% (Valle d'Aosta) (see Supplementary Table S4).

#### 3.1.1. Results of univariate meta-analyses and meta-regressions

Table 1 summarizes the results of the Bayesian meta-analyses on the quantities derived from the regional *R*_0_(*t*) curves: average value of *R*_0_(*t*) from October to December, maximum value of *R*_0_(*t*) over the study period, changes in *R*_0_(*t*) arising in 4 weeks after school re-opening and after the introduction of national restrictions on November 6th. For all quantities, the heterogeneity among regions was high, with the lowest *I*^2^ index estimated among the Southern regions (the *I*^2^ index is the percentage of the total variability due to the between regions heterogeneity—see Supplementary Section S5). The average level of *R*_0_(*t*), as well as its maximum, was higher in the Central and Northern regions than in the Southern ones. An overall increase of *R*_0_(*t*) equal to 0.50 was estimated during the first 4 weeks after school re-opening in September (the dates of school re-opening in every region are reported in Supplementary Table S5). This increase was lower in the Southern regions than in the Central and Northern ones.

**Table 1:** Results of the Bayesian meta-analyses (posterior mean of the quantity of interest and posterior median of the *I*^2^ index, with associated 90% credible intervals) conducted on: average value of *R*_0_(*t*) from October to December, maximum value of *R*_0_(*t*) over the study period, changes in *R*_0_(*t*) after school re-opening and after the introduction of national restrictions on November 6th; *p* = 1.15%, *T* = 14 days.

		**Estimate**	**90% CrI**		***I* ^2^**	**90% CrI**	
Average *R*_0_(*t*) from October to December	All regions	1.59	1.53	1.65	90.2	83.0	94.7
	North	1.66	1.57	1.76	92.1	82.0	97.4
	Center	1.61	1.44	1.78	93.1	79.6	98.6
	South	1.48	1.39	1.56	80.2	56.5	94.0
Maximum *R*_0_(*t*) over the study period	All regions	2.61	2.39	2.83	91.6	84.8	95.7
	North	2.83	2.44	3.26	94.5	85.0	98.3
	Center	2.78	2.27	3.32	87.5	65.2	97.5
	South	2.15	1.97	2.35	55.3	20.3	88.9
4 week change in *R*_0_(*t*) after school re-opening	All regions	0.50	0.35	0.66	87.5	78.0	93.3
	North	0.74	0.54	0.94	74.4	32.5	93.0
	Center	0.58	0.15	1.03	89.7	69.9	98.0
	South	0.16	0.02	0.30	55.7	24.6	86.1
4 week change in *R*_0_(*t*) after November 6th	All regions	-0.75	-0.84	-0.66	88.4	80.9	93.7
	North	-0.72	-0.91	-0.54	95.8	90.4	98.7
	Center	-0.73	-0.90	-0.56	76.7	46.6	94.8
	South	-0.81	-0.97	-0.66	76.3	45.0	93.1

**Figure 4:**
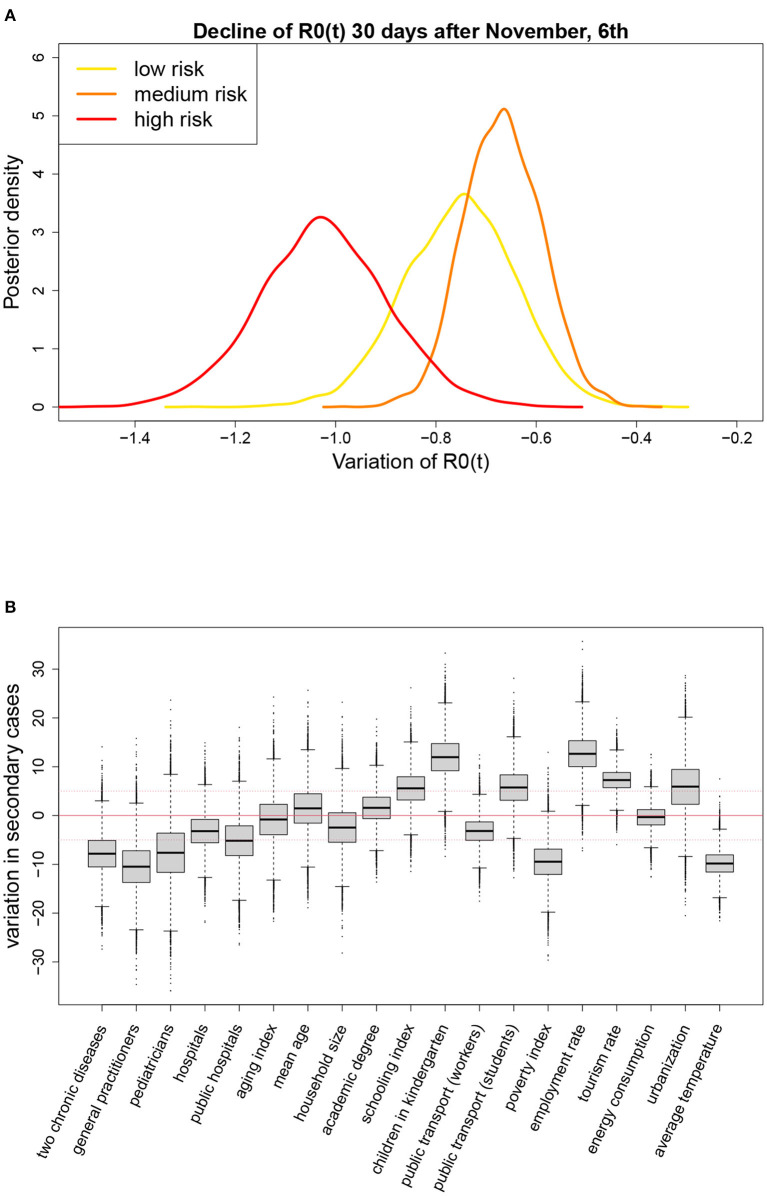
**(A)** Posterior distributions of the change in *R*_0_(*t*) during the first 4 weeks following the introduction of the restriction measures on November 6th, by level of alarm assigned to the region, and **(B)** results of the meta-regressions on the average value of *R*_0_(*t*) from October to December (posterior distribution of the variation in the average value of *R*_0_(*t*) ×100, associated to a change in the meta-regressor equal to its observed interquartile range); *p* = 1.15%, *T* = 14 days.

We found also evidence of an overall decline of *R*_0_(*t*) equal to 0.75 during the 4 weeks following the introduction of the restrictions on November 6th. This decline was similar across geographical areas (Table 1), but appeared to be associated with the strength of the measures adopted at a regional level, as shown by the posterior distributions reported in Figure 4A. In the four regions initially classified as at high risk (red regions: Calabria, Lombardia, Piemonte, Valle d'Aosta), where stronger restrictions were immediately adopted, the decline was steeper than in the two regions classified as at medium risk (orange regions: Puglia, Sicilia) and in the remaining 14 low risk regions (yellow), subject to lighter measures.

Figure 4B and Supplementary Table S6 show the results of the meta-regressions on the average value of *R*_0_(*t*) from October to December. For each meta-regressor, the result is reported in terms of change in the average value of *R*_0_(*t*) associated with a variation of one interquartile range (IQR) in the meta-regressor itself. The change was multiplied by 100 so that the reported value indicates the change in the number of secondary infections derived from 100 infected individuals. For example, we found that an increase of 6.75 in the percentage of students that used public transport—6.75 is the IQR for this meta-regressor reported in Supplementary Table S6—was associated with an increase of 5.76 units in the average number of secondary infections derived from 100 infected subjects, or that an increase of 1.14 in the number of family physicians per 10'000 residents was associated with a 10 units decrease in the average number of secondary infections derived from 100 infected subjects. We considered a meta-regressor relevant if the posterior probability that the change was larger than 5 or lower than -5 exceeded 50%, i.e., if the posterior median, represented by the horizontal line through the box in Figure 4B, was outside the range [−5, 5] (notice that median and mean are very close given the symmetry of the posterior distributions). According to this criterion, it was evident a positive marginal association of the average *R*_0_(*t*) with employment rate, use of kindergartens, tourism rate and, to a less extent, with the percentage of population living in high urbanization areas, schooling index, and use of public transport to go to school. A negative association was found with the number of practitioners and pediatricians per inhabitant, poverty index, temperature, prevalence of people with at least two chronic diseases, and, to a less extent, with the proportion of public hospitals.

### 3.2. Results of the sensitivity analyses

**Figure 5:**
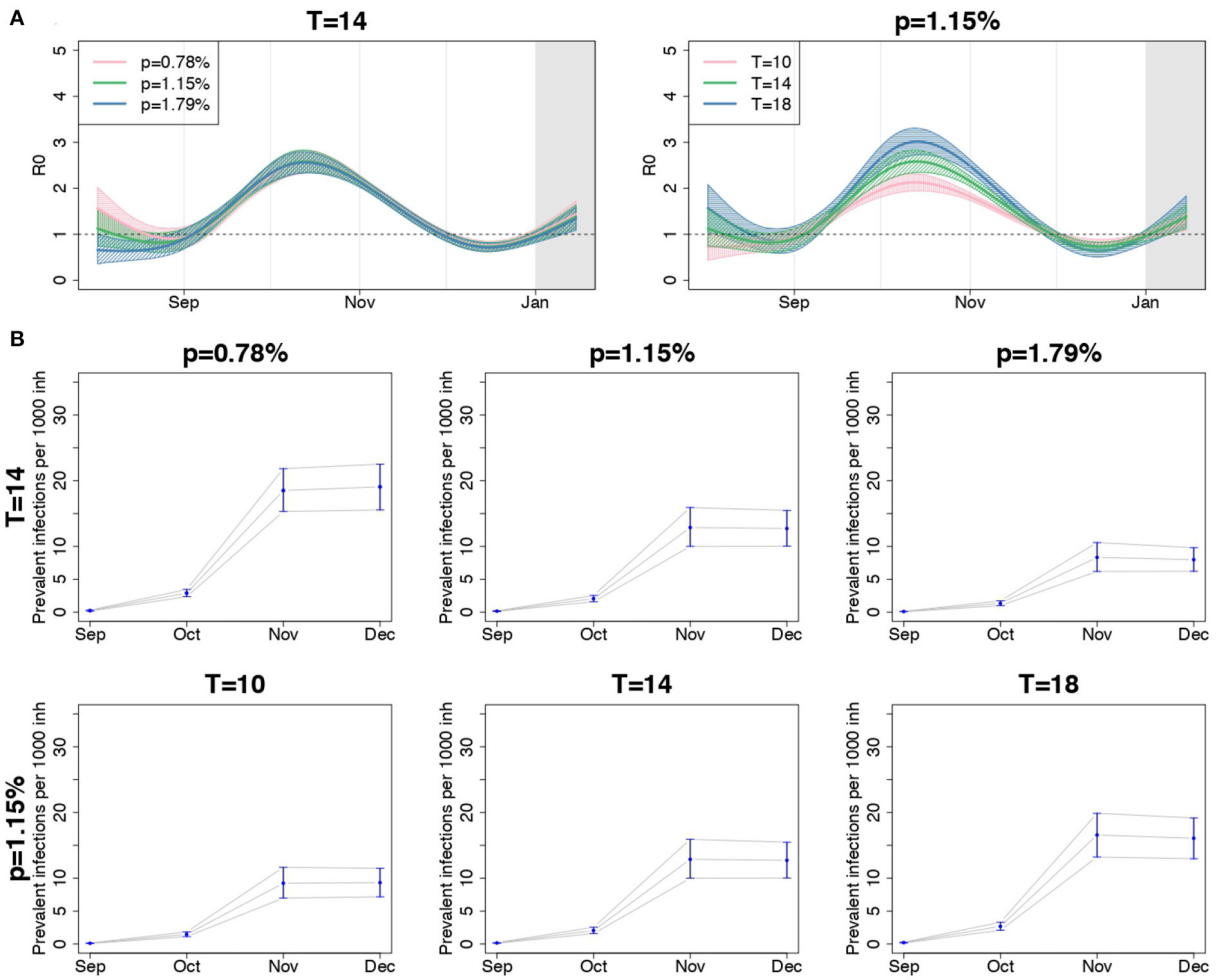
**(A)** Posterior mean and 90% credible bands of the meta-analytic *R*_0_ curves and **(B)** posterior mean and 90% credible intervals of the meta-analytic monthly-averaged prevalence per 1,000 inhabitants (second and third rows) for the entire country, when using different values for *p*, fixed *T* = 14 days, and when using different values for *T*, fixed *p* = 1.15%.

The shape of the regional *R*_0_(*t*) curves appeared quite similar under different IFR scenarios when *T* = 14, as shown by the comparison of the overall meta-analytic curves obtained by fixing the IFR to different values in the regional SIRD models (left panel of Figure 5A). Some discrepancy was observed only at the beginning of the study period, when larger values of *R*_0_(*t*) were obtained in correspondence of smaller values of *p*. The regional curves obtained changing *p* are shown in Supplementary Figures S5–S7.

The right panel of Figure 5A compares the overall meta-analytic curve obtained by assuming different values for *T*, having fixed *p* = 1.15%. A shorter infection duration corresponded to a less peaked curve, but the date when *R*_0_(*t*) was maximum and the date when it first crossed the value of 1 after the introduction of containment measures were preserved.

In the lower panel of Figure 5, we reported the meta-analytic estimates of the monthly averaged prevalence of infections per 1,000 inhabitants from September to December, when changing *p*, fixed *T* (second row), and when changing *T*, fixed *p* (third row). The estimated prevalence decreased consistently with the value of *p* and increased with the values of *T*. Notice that the number of prevalent infections when *T* = 10 and *p* = 1.15% and when *T* = 14 and *p* = 1.79% resembled one another. The same happened with the pairs of parameters *T* = 14, *p* = 0.78%, and *T* = 18, *p* = 1.15%. This suggests that increasing (decreasing) *p* produces the same effect of decreasing (increasing) *T*, when the other parameter is fixed, and that a global evaluation of the impact of these quantities on the results is required.

Table 2 shows the total variance indexes, including the aggregate index for the vector ***ϑ***, and the first order indexes obtained from the GSA procedure. As suggested by the magnitude of the total variance indexes, changes of the fixed parameters *p*, *T*_*D*_, and *I*(0) had a very low impact on the coefficients vector ***ϑ***. On the contrary, *T*_*R*_ exhibited the greatest aggregated total variance index (0.72), proving to be an input that contributed very much to the *R*_0_(*t*) curve as a whole. Regarding the impact on the derived quantities, *T*_*R*_ was still the most relevant input (the only relevant on the maximum values of *R*_0_(*t*)), even if non-negligible indexes were found also for the other inputs, especially on the date of occurrence of the maximum *R*_0_(*t*), on the date when *R*_0_(*t*) crossed the threshold of 1 and on the date of the peak of infections.

**Table 2:** Total variance indexes and first order indexes of each model input (by row) on the coefficients of the *R*_0_(*t*) regression spline, maximum and minimum values of *R*_0_(*t*) with the corresponding dates, date in which *R*_0_(*t*) first crossed the value of 1, infection peak with the corresponding date (by column).

**Total variance indexes**														
**Spline coefficients**								*R*_0_(*t*)					**Infections**	
	ϑ_1_	ϑ_2_	ϑ_3_	ϑ_4_	ϑ_5_	ϑ_6_	**Aggr**. ***ϑ***	**Max**	**Max**	**Min**	**Min (date)**	**Cross 1 (date)**	**Peak**	**Peak (date)**
*p*	0.10	0.21	0.12	0.08	0.08	0.21	0.09	0.02	0.39	0.19	0.14	0.16	0.16	0.50
*I*(0)	0.18	0.35	0.23	0.16	0.14	0.36	0.17	0.02	0.58	0.27	0.19	0.03	0.00	0.45
*T*_*D*_	0.18	0.40	0.21	0.15	0.14	0.34	0.17	0.03	0.73	0.30	0.20	0.18	0.32	0.57
*T*_*R*_	0.68	0.10	0.61	0.72	0.78	0.23	0.72	0.99	0.72	0.69	0.78	0.87	0.74	0.85
**First order indexes**														
**Spline coefficients**								*R*_0_(*t*)					**Infections**	
	ϑ_1_	ϑ_2_	ϑ_3_	ϑ_4_	ϑ_5_	ϑ_6_		**Max**	**Max (date)**	**Min**	**Min (date)**	**Cross 1 (date)**	**Peak**	**Peak (date)**
*p*	0.07	0.20	0.09	0.07	0.05	0.12		−0.01	−0.06	0.10	0.03	0.01	0.04	0.14
*I*(0)	0.12	0.34	0.16	0.11	0.08	0.32		0.00	0.00	0.13	0.02	−0.04	−0.04	0.05
*T*_*D*_	0.11	0.32	0.13	0.09	0.07	0.21		−0.03	−0.26	0.14	0.06	0.00	0.11	0.02
*T*_*R*_	0.53	0.03	0.43	0.60	0.64	0.09		0.95	−0.18	0.42	0.61	0.73	0.56	0.25

In order to correctly interpret these results, one should however consider that some outputs could vary within a small range of values as the inputs change. Hence, the corresponding total variance indexes, although high, could actually derive from the apportionment of a small total variance. This is in itself indicative of overall robustness of these outputs to inputs perturbations. For instance (Supplementary Figure S8 and Supplementary Table S7), the date of the infection peak (coefficient of variation 0.002) was almost unaffected by variations of the model inputs as well as the date in which *R*_0_(*t*) crossed the value of 1 (coefficient of variation 0.01). Analogously ϑ_4_ had the most dispersed distribution among the other coefficients, suggesting its greater sensitivity to changes of the inputs values. This larger dispersion was considered in the calculation of the aggregate total variance index, which averages the single total variance indexes with weights proportional to the ϑ_*i*_ variances (36).

While in the regional SIRD models we constrained *T*_*D*_ = *T*_*R*_ = *T*, the GSA was conducted allowing *T*_*D*_ to vary independently of *T*_*R*_. This choice emphasized that *T*_*D*_ was less relevant than *T*_*R*_ on output variability. Therefore, we can conclude that, when *T*_*R*_ is properly set, misspecification of *T*_*D*_ has a negligible effect. In such a case, forcing *T*_*D*_ to be equal to *T*_*R*_, as in our regional analysis, produces a simplification of the SIRD model without inducing relevant variations in the outputs.

Finally, being the total variance indexes for ***ϑ*** only slightly higher than the corresponding first order indexes, it was evident that interactions among inputs were not relevant on the estimated *R*_0_(*t*) curve. On the contrary, interactions were a relevant source of variability in the derived quantities.

The fact that the first order indexes on the derived quantities were sometimes negative, in most cases close to zero, was due to a poor MC approximation. Negative indexes are not unusual when the contribution of the inputs is negligible and can be avoided by increasing the number of MC simulations (20).

### 3.3. Comparison between observed infections and infections predicted by the model

The number of new infections estimated by the SIRD model should be interpreted as inclusive of the undetected cases, thus one would expect it to be an upper bound for the observed number of new notified cases reported by the Protezione Civile. However, for some regions the observed number of new notified infections sometimes exceeded the number of new infections estimated by calibrating the SIRD model on the observed COVID-19-related deaths (Supplementary Figure S3). This paradoxical result could be partly due to systematic errors (e.g., notifications concentrated on particular days of the week) and delays in the notification of cases but it could be also related to an inappropriate assumption on *p*. In fact, as the lower panel of Figure 5B shows, the predicted number of infections strongly depends on the value of *p* assumed in the model. In other words, given the observed time series of COVID-19-related deaths, we could have estimated a lower prevalence of infections assuming a higher IFR scenario, and conversely a higher prevalence of infections assuming a lower IFR scenario. Accounting for this, if the observed number of new notified infections exceeds the estimated number of new infections, the value *p* = 1.15% could be too high.

**Figure 6:**
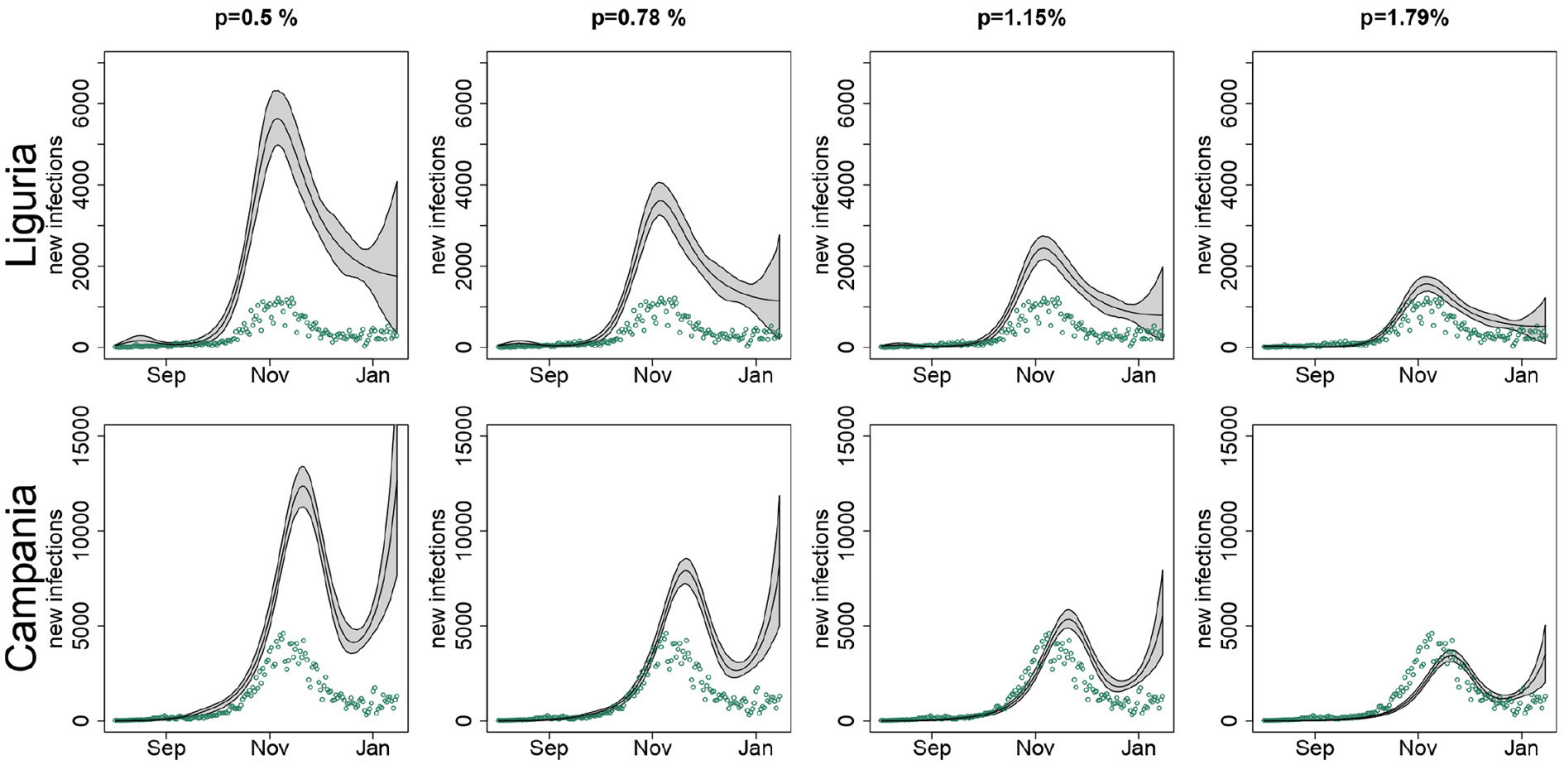
Estimated number of new infections (black line) in Liguria and Campania with pointwise 90% confidence bands, when setting in the SIRD model the infection fatality rate *p* to 0.5, 0.78, 1.15, and 1.79%, along with the observed number of new notified infections (green points), by region; *T* = 14 days.

As an example, in Figure 6 we compared the predicted number of new infections for Campania and Liguria under different IFR scenarios. As the value assumed for the IFR increased, the number of new infections estimated by the model became smaller, to the extent of being, in the case of Campania, inconsistently lower than the observed number of new notified infections when *p* = 1.15% and *p* = 1.79%. On the contrary, for Liguria the estimated curves were overall consistent with the observed number of new notified infections, regardless of the IFR used in the analysis. The only exception was for *p* = 1.79%, when the observed number of new cases notified during August and September slightly exceeded the model predictions.

## 4. Discussion

In this paper, we used official data publicly available to study the SARS-CoV-2 epidemic dynamics in Italy and to investigate regional heterogeneity. We conducted our analyses on the time window corresponding to the second epidemic wave in Italy, which did not include the first months of 2021, when an extensive vaccination campaign began in the country. This permitted us to specify regional models that did not account for the immunity progressively acquired by part of the population.

In the literature a variety of indexes describing the evolution of the pandemic have been proposed (see Giraudo et al. (37) among others). However, here we focused only on the instantaneous reproductive number, *R*_0_(*t*), and we modeled it through a regression spline in order to capture how the strength of contagion varied over time. *R*_0_(*t*) describes the speed of the contagion and depends directly or indirectly on countless factors, including virus infectiousness, socio-demographic and economic characteristics of the population, the efficacy of contact-tracing procedures, restriction policies. Being directly related to the number of contacts across the population, it is very sensitive to the introduction of social distancing measures.

Alternative methods exist to estimate *R*_0_(*t*) (25), but the main advantage of estimating *R*_0_(*t*) through compartmental models is that they allow quantifying also the number of incident and prevalent infections over time, and, more in general, the size of all defined compartments. Additionally, they permit to estimate *R*_0_(*t*) relying on the observed time series of daily COVID-19-related deaths, which, in our context, was the most reliable quantity among those reported in the Protezione Civile database. Even though we cannot exclude a certain amount of mortality under-reporting—an important problem during the initial phase of the COVID-19 emergency—this was probably negligible during the second epidemic wave.

Under the hypothesis that the assumptions underlying the SIRD model are acceptable, the good predictive performance of our approach suggests that we are correctly describing the variations of the infectious reproductive number over time.

### 4.1. Discussion on the main analysis

Our results indicated that in Italy, during the second SARS-CoV-2 epidemic wave, the instantaneous reproductive number changed over time heterogeneously across regions, but with some important common elements. Among them, the increase of *R*_0_(*t*) during September, stronger in the Northern and Central regions than in the Southern ones, which led to a dangerously high level of contagion in mid-October, followed by a decline of the contagion spread. We cannot exclude that this pattern was due to a phenomenon of seasonal variation typical of respiratory infections (38), but it was also suggestive of a possible role of school reopening in September in amplifying infection spreading, as argued also by Larosa et al. (39). Although contagion within school has been sometimes declared to be no higher than in other contexts (40, 41), this issue is still debated (42). In particular, reopening schools may have facilitated contagions through intensifying the number and the duration of interpersonal contacts within and outside schools, especially with the use of the public transport (1). This seems to be confirmed by the meta-regression results: the regions where the percentage of students using public transport was higher tended to be characterized by higher average levels of *R*_0_(*t*).

Also meteorological conditions (temperature, humidity and UV radiations) may have had an impact on contagion spread through modulating SARS-CoV-2 infectiousness (38, 43–45). Additionally, with the arrival of the autumn, recreational and sport activities moved, as usual, to closed places, with a consequent increase of the contagion risk (46, 47). The possible role of ambient temperature as a moderator of the contagion was suggested also by the meta-regression: higher average regional temperatures were associated with lower values of the average *R*_0_(*t*) from October to December. One factor that may have contributed to the peak of the *R*_0_(*t*) curve in October was also the full resumption of many work activities after the summer vacation.

Finally, at the end of September (20th-21st) regional elections took place in seven regions (Toscana, Marche, Campania, Puglia, Veneto, Liguria, Valle d'Aosta) and administrative elections took place in 1,184 municipalities around the country. The fact that, as per tradition, voting stations have been set up in school buildings during the weekend and that people moved within and between regions to reach their places of residence in order to vote should not be a priori ruled out as a possible source of contagion amplification, as documented elsewhere (48, 49).

The decline of the *R*_0_(*t*) curves after the October peak was likely related to the restrictions progressively put in place by the central government. This decline tended to be steeper in the regions earlier classified as high-risk zones, where stronger restrictions were immediately introduced. However, it should be also noticed that, especially in the Northern and Central regions, the decline apparently started before the introduction of restrictions on November 6th. This could be indicative of the efficacy of local measures introduced in some regions before the national ones or reflect spontaneous changes in behavior of the population in the face of worrying levels of contagion. At the end of November, the instantaneous reproductive number was below or very close to the threshold of 1 everywhere. Then, a new increase was observed during the second half of December. This was particularly evident in the Southern regions, probably due to travels within and between regions related to the Christmas holidays.

Overall, our results about the shape of *R*_0_(*t*) are coherent with those reported in other analyses of the second epidemic wave in Italy. In particular, Ferrari et al. (10), who estimated the reproductive number at the provincial level by calibrating on notified cases, found that by mid-October the reproductive number was greater than 2 in almost all provinces. Furthermore, in many provinces of Liguria, Abruzzo, Toscana and Umbria they estimated a reproductive number consistent with our finding that in these regions *R*_0_(*t*) reached the value 1 faster than in others (Figure 1). Finally, similarly to us, they conclude that in December the reproductive number was smaller than 1 in almost all of Italy.

The “flat” *R*_0_(*t*) curve estimated for the Southern regions, with a maximum value slightly larger than 2, versus a maximum close to 3 in the rest of the country, was indicative of apparently different dynamics of contagion in the South, perhaps related to specific environmental, demographic, and socioeconomic characteristics or to a greater ability to control contagion. Focusing on the average level of *R*_0_(*t*), we tried to explain through meta-regression part of the observed between-region heterogeneity. Our meta-regression results should not be used to draw conclusions about the existence of causal relationships linking the strength of contagion with the regional features, but suggest hypotheses that should be investigated through subsequent *ad-hoc* studies. The positive association of the average value of *R*_0_(*t*) with employment rate and schooling rate, and its negative association with the poverty index seems to indicate that population lifestyles typical of richer socio-economic contexts may have helped the virus spread. Also, population density, here measured in terms of percentage of people residing in high urbanization areas, was one of the possible predictors of a higher instantaneous reproductive number, as well as tourism attractiveness of the region. However, in interpreting this latter result, one should consider that tourism flows have undergone considerable changes due to the COVID-19 emergency and that in our meta-regression we included the tourism rate of the year 2018, the last available.

As already discussed, our meta-regressions highlighted the potential role of school-related commuting in enhancing the contagion. We also found that the percentage of children attending kindergarten had a positive association with the *R*_0_(*t*) level. This result could simply reflect the fact that taking advantage of the kindergartens service is a proxy of the employment level in the region or could be indicative that kindergartens themselves have had a role in spreading infections. Notice that kindergartens have been usually kept open during the second epidemic wave. Interestingly, we found that the average *R*_0_(*t*) was lower where the number of family physicians and pediatricians per inhabitants, as well as the proportion of public healthcare institutes, were higher. These results could indicate the important role of family medicine and public sanitary service in preventing contagion.

In interpreting the results of the meta-regression, we must take into account that some of the meta-regressors followed a North-South gradient, so it is difficult to disentangle their role from that of possible unobserved factors that varied with latitude. However, it is also worth noting that our meta-regression results are very similar to those reported elsewhere and obtained using analytical approaches different from ours (e.g., regressions and geographical modeling) on data from different countries. There are several studies in the literature that have explored the association of socioeconomic, demographic, and health variables with COVID-19-related cases and deaths (50–56). Chang et al. (55) analyzed the role of variables measured before the COVID-19 outbreak in explaining the number of confirmed infections and deaths in 91 countries during the second epidemic wave. Similarly to us, they list as "aggravating" factors the level of urbanization, the average age of the population, tourism, and indicators of economic well-being such as GDP; temperature and health infrastructure are listed among the "mitigating" factors, as well as, unlike in our analysis, the level of education of the population. In line with us, most studies have found a positive association between infection level and population density (50–54) and, interestingly, Gonzalez-Val et al. (50) report a negative association between the number of infections and physician density. Sã (54), focusing on the early months of the COVID-19 epidemic in England and the Wales, found that areas where there was a higher fraction of people using public transport had a higher number of COVID-19 infections per 100,000 people. Our result on the percentage of children attending kindergarten is not confirmed in González-Val and Sanz-Gracia (50), where a negative association is reported between the number of cases and the percentage of schoolchildren and children attending daycare.

Since the onset of the COVID-19 emergency, interest on the relationship between contagion and weather conditions has been very strong (57, 58). Several studies conducted on a global scale and on specific countries focused on the association between climate and the spread of the SARS-CoV-2 virus (59–61). In most cases, the evidence indicated that the level of contagion was lower where temperatures were higher. Our meta-regression results are consistent with this literature.

### 4.2. Discussion on the sensitivity analyses

Compartmental models can be very complex when many compartments are defined and many transitions between them are allowed. Complexity goes hand in hand with an increase in the number of unknown parameters that cannot be estimated due to structural and practical identifiability problems. Most of the complicated compartmental models proposed in the literature rely on fixing the values of a large number of parameters without even studying the impact on the results of those arbitrarily chosen values (21, 62).

In this paper, we performed both one-factor-at-a-time sensitivity analysis and GSA. These two sensitivity analyses have a different interpretation and their results were not directly comparable. In fact, in the one-factor-at-a-time sensitivity analysis we ignored how the inputs interacted and inspected the model outputs only for a few values of IFR and infection duration. On the contrary, with the GSA, we explored the whole space of the non-estimated parameters of the SIRD model.

The GSA indicated that there was interaction among the inputs and that there were no completely negligible inputs. The initial number of infected *I*(0) resulted to be the less influential input. Notice that we were interested in evaluating the relevance of *I*(0) since in the main analysis we forced it to be equal to the number of notified infections present in the region on July 31st, thus assuming that the regional screening and tracing systems were initially able to detect all new infections. A second important result was related to the model simplification that we adopted in the regional SIRD models, assuming *T*_*D*_ = *T*_*R*_ = *T*: this simplification is admissible if the infection duration is set to plausible values for *T*_*R*_. On the other hand, *T*_*R*_ resulted to be the most influential parameter, suggesting that an accurate knowledge of *T*_*R*_ would be needed for better predictions and understanding of the pandemic. Unfortunately, improving the empirical evidence on *T*_*R*_ is not trivial, because of its complex nature (*T*_*R*_ is both the average time from infection onset to recovery and the average time of infectiousness) and its dependence on undetected infections.

Finally, the GSA confirmed the robustness, already documented elsewhere (29), of the estimated *R*_0_(*t*) curve to the value assigned to *p*. Under the reasonable assumption that the average infection time was homogeneous across the regions (the infection time is mainly related to virus and disease characteristics) and considering that changing the value of *T* the regional *R*_0_(*t*) curves inflated/deflated but preserved their overall shape, this robustness should assure that the conclusions drawn from the comparison of the regional *R*_0_(*t*) curves are not affected by specific choices of IFR and *T*.

### 4.3. Discussion on the IFR value

Our model produced regional estimates of the number of circulating infections, inclusive of the submerged fraction of the contagion. This quantity, combined with *R*_0_(*t*), determines the number of new infections, and it is useful to assess the actual impact of the contagion on the healthcare system: if a large instantaneous reproductive number can be sustainable at the beginning of the epidemic when the number of cases is low, a low instantaneous reproductive number may not be sustainable when the number of infections is large.

For identifiability reasons, we did not estimate the IFR, but our analysis indirectly provided indications about plausible values for this parameter through a comparison between the observed cases reported by the Protezione Civile (18) and the number of infections predicted by the SIRD model under three different scenarios of IFR. Under the assumption of an average waiting time from infection onset to infection resolution (death or recovery) equal to 14 days, our findings seem to indicate that during the study period the IFR may have changed over time and space. In particular, according to our results, the IFR was likely lower than 1.15% in the initial phase of the second epidemic wave, when the virus mainly circulated among younger people (63), as well as in many Southern regions, including Campania, which is, not by chance, the region with the lowest aging index in Italy (22). Conversely, an IFR equal to 1.79%, the upper bound considered in our sensitivity analysis, is not consistent with the observed infection dynamics in most regions, because it would lead to a predicted number of infections smaller than the observed number of notified cases.

Assuming *p* = 0.5%, not far from the IFR estimate reported in the review by Ioannidis (64), we would obtain quite high estimates of infection prevalence which could be reasonably considered as an extreme—even though not impossible—upper bound.

Notice that, in principle, the observed inconsistency between predicted infections and notified infections in the Southern regions could partly be solved by assuming for them a longer average waiting time *T* (e.g., *T* = 18 days), with *p* = 1.15%. Indeed, as shown by the sensitivity analysis results, the parameters *p* and *T* jointly affect the epidemic curve generated by the SIRD. However, as already explained, this hypothesis seems less plausible than the hypothesis of a heterogeneous IFR.

### 4.4. Study limitations

Our study has some limitations. Regarding the regional analyses, the SIRD model relied on strong assumptions that could be partly unrealistic: the population was homogeneously mixed, with people making contact at random, and closed, with no contacts among individuals belonging to different regions or countries; transition parameters were constant across individuals who were present at the same time in the same compartment; there was not reinfection and no incubation period. Importantly, an individual becoming infected on the day *t* was supposed to be infectious starting from the day *t*+1 until infection resolution. This was quite an unrealistic assumption for the infected individuals that were notified, thus isolated. Additionally, as usual in compartmental models in their simplest form, we implicitly assumed that the transition times were exponentially distributed (65), inducing a non-negligible probability of infection durations much longer than the average, as well as a high probability of very short waiting times.

As already discussed in Section 4.3, also the assumption of an IFR which is constant over time was questionable, in the light of the comparison between the observed notified cases and the infections predicted by the SIRD model. In fact, the IFR may have changed over time due to the spread of new variants with different lethality, as well as to changes in the composition of the population at risk that may have become on average more or less frail over time.

We calibrated the SIRD models only on the observed COVID-19-related deaths, without exploiting the availability of other observed time series, like the ones of notified infected. Calibrating on the notified infected would have required the formulation of a more complex compartmental model with separate compartments for detected and undetected cases and the introduction of additional unknown transition parameters (2–4, 29). On the other hand, this more complex model would have also allowed us to take into account the fact that, once detected and isolated, the infected individuals spread the contagion less than the undetected ones. An additional limitation concerns the fact that the regression spline used to model the regional *R*_0_(*t*) curves could be quite sensitive to the knots position. One could extend the procedure to the use of penalized splines (26).

Following a procedure which is frequently used in compartment models literature (32, 66), we did not make assumptions about data distribution in the phase of estimation of the SIRD parameters, but we generated bootstrap samples for confidence intervals construction by assuming a Negative Binomial distribution on the daily increments of deaths. Alternative approaches rely on likelihoods maximization or Bayesian inference, but compartmental models often exhibit complex likelihoods requiring particle filtering methods to be maximized (67) or computationally intensive methods based on data augmentation procedures (68). In the Bayesian framework, likelihood-free approaches have been also proposed (69).

The bootstrap percentile intervals could have lower coverage than the nominal one (70). Bias-corrected and accelerated bootstrap confidence intervals could have better performance (71), but they are too computationally demanding to be applied in this context.

In meta-regression we did not focus on environmental variables other than the regional average temperature, but also solar radiation, humidity, and air pollution have been suggested as affecting the contagion rate as well as COVID-19-related mortality or hospitalization (72–74). Finally, a finer geographic detail, such as considering provinces or municipalities instead of regions, would have allowed us to better appreciate the sources of heterogeneity in the spread of contagion. However, besides the fact that in Italy mortality data are not made freely available at this detail, estimating *R*_0_(*t*) at the provincial or finer level would have led to a more unstable inference. Not to mention that, at this geographic detail, the assumption of a closed population would be even more questionable.

## 5. Conclusion

Despite the difficulty of drawing information from limited data, our approach allowed us to estimate *R*_0_(*t*) and to evaluate the prevalence of infections during the second SARS-CoV-2 wave in Italy at the regional level.

Beyond the common elements—including a peak in mid-October and a decline during November, which was more pronounced in regions where stronger restrictions were applied first—the rate of contagion changed heterogeneously among regions over time. This shows that models treating the phenomenon at the national level could ignore important characteristics, specific to certain areas.

The meta-regression results show that the observed heterogeneity can be partly explained by socioeconomic and demographic factors, such as level of urbanization, family medicine and healthcare system, employment rate, use of public transport for school commuting. Higher temperatures were associated to lower *R*_0_(*t*) levels. These factors should be further explored with finer geographical scale analyses.

The results of the sensitivity analyses reassure us that the overall shape of the estimated *R*_0_(*t*) curves and the conclusions drawn from the comparisons of the regional curves are robust to changes in the values of the parameters that we considered as fixed to make the SIRD model identifiable. On the contrary, the estimate of the prevalence of infections was strongly influenced by the assumptions regarding the IFR. Furthermore, our results seem to support the hypothesis of a heterogeneous IFR over time and across regions, that may have been lower than 1% in some regions, especially at the beginning of the epidemic wave.

Regarding sensitivity analysis, GSA has proven to be a powerful and promising tool and its use should be encouraged in this and other research contexts.

## Data availability statement

This study is based on publicly available datasets. The data used in the compartmental models are available here: https://github.com/pcm-dpc/COVID-19.

## Author contributions

MB supervised the project. All authors conceived the study, performed the statistical analyses, discussed the results, and wrote the paper. All authors contributed to the article and approved the submitted version.

## Funding

This work was partly conducted within a Research Agreeement between Department of Statistics, Computer Science, Applications, University of Florence and Agenzia Regionale di Sanità della Toscana (ARS-REP 2321/2020 “SARS-CoV-2 epidemic. Analytical tools for modeling and predicting contagion dynamics and their impact on the health service”, Responsible: MB).

## Conflict of interest

The authors declare that the research was conducted in the absence of any commercial or financial relationships that could be construed as a potential conflict of interest.

## Publisher's note

All claims expressed in this article are solely those of the authors and do not necessarily represent those of their affiliated organizations, or those of the publisher, the editors and the reviewers. Any product that may be evaluated in this article, or claim that may be made by its manufacturer, is not guaranteed or endorsed by the publisher.
