# Supplementary material for: Combining and comparing regional SARS-CoV-2 epidemic dynamics in Italy: Bayesian meta-analysis of compartmental models and global sensitivity analysis
Source: Front Public Health. 2022 Sep 16;10:919456. doi: 10.3389/fpubh.2022.919456 (PMC9523586; doi:10.3389/fpubh.2022.919456)
Supplement: Supplementary file 1 [file Data_Sheet_1.pdf]

Supplementary Material

FIGURES

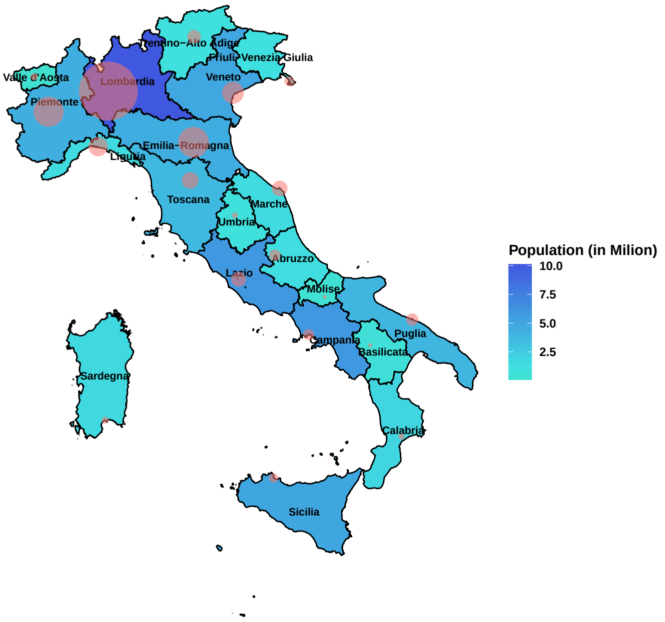

Figure S1. Italian regions colored according to the population size, with bubbles of diameter proportional to the number of COVID19-related deaths observed from August 1st 2020 to January 14th 2021 (Protezione Civile)

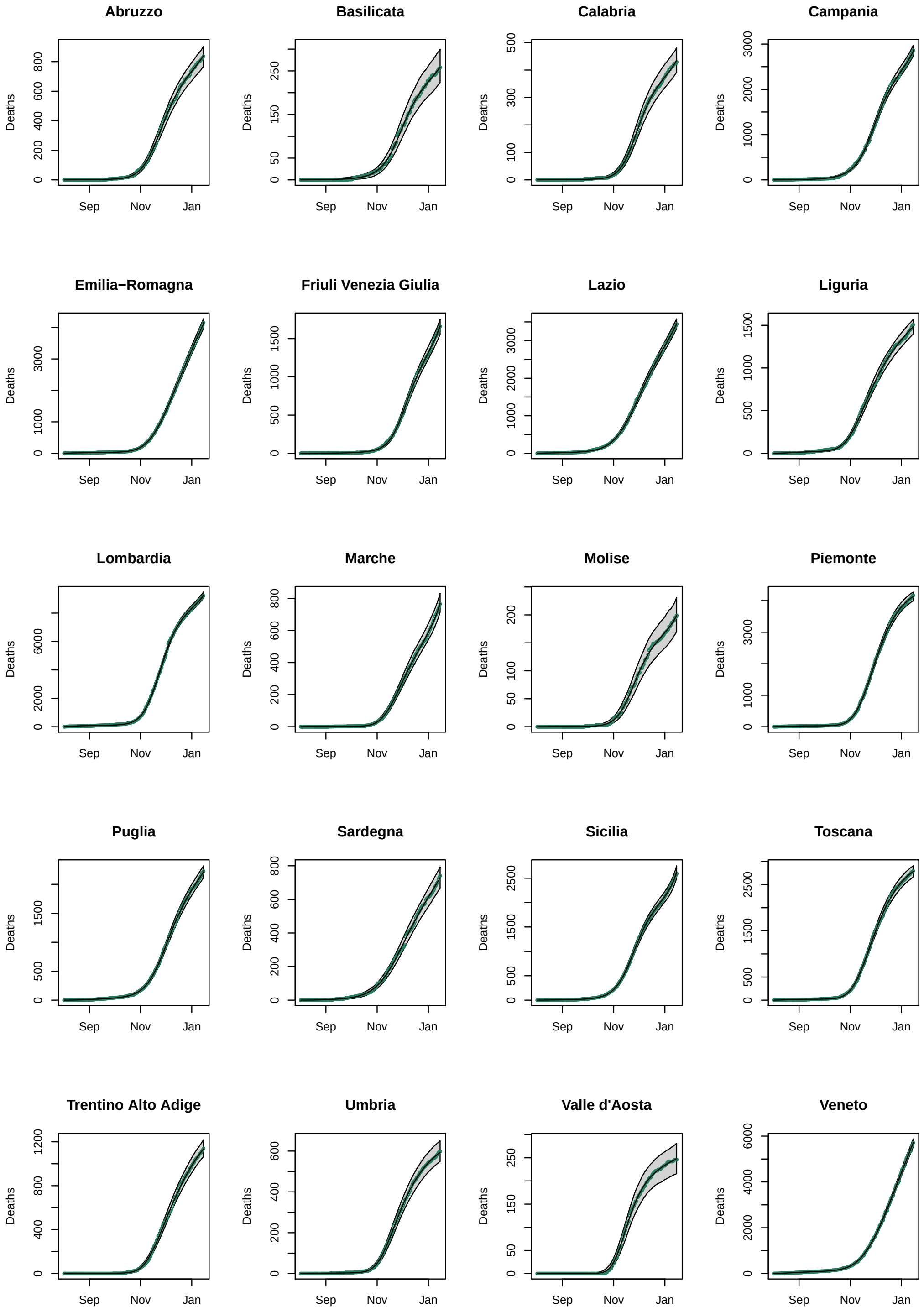

Figure S2. Estimated number of COVID19-related deaths with pointwise 90% confidence bands and observed number of COVID-19 deaths (green points), by region; $p = 1.15\%$, $T = 14$ days.

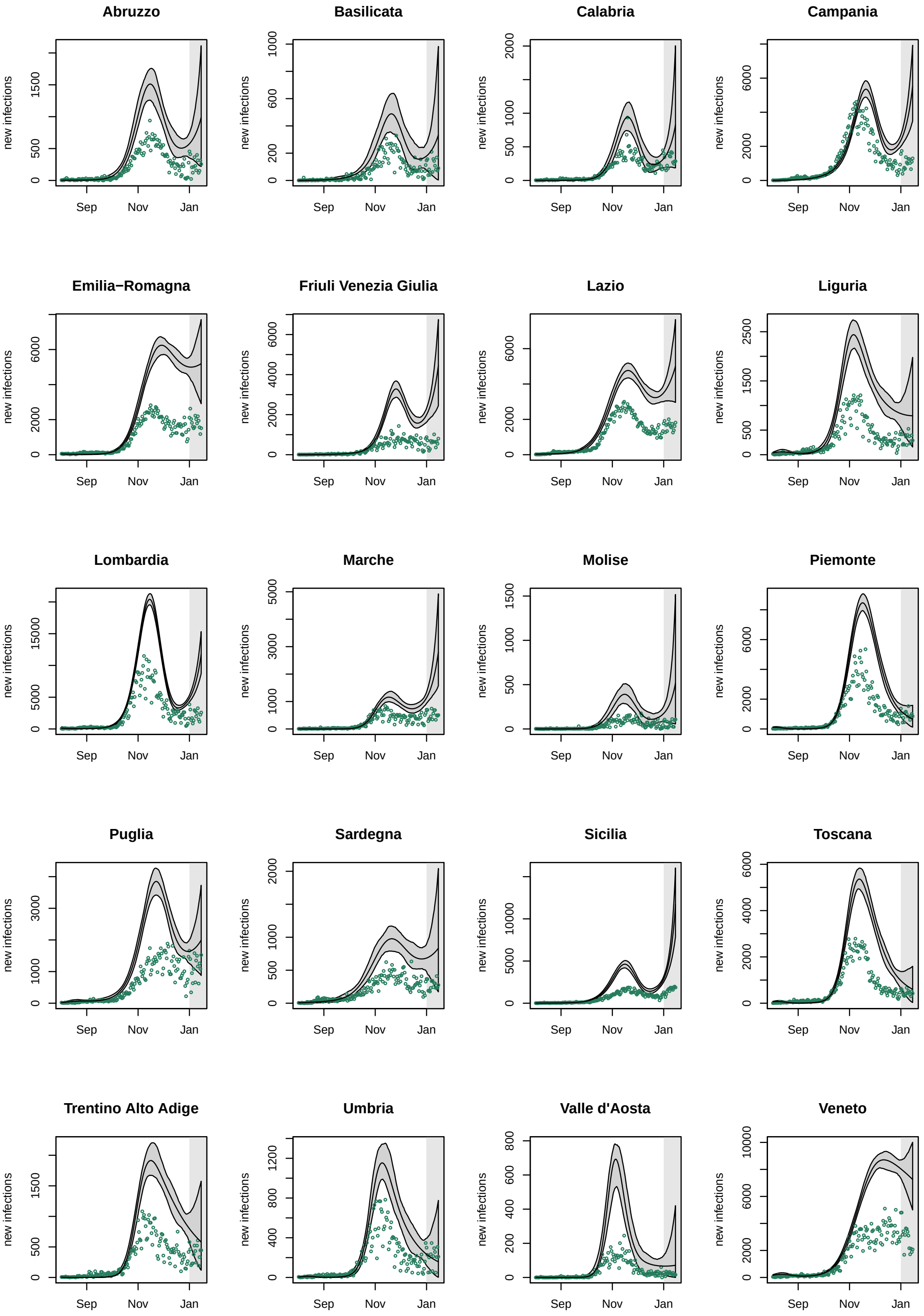

Figure S3. Estimated number of new infections with pointwise 90% confidence bands and observed number of new notified infections (green points), by region; $p = 1.15\%$, $T = 14$ days.

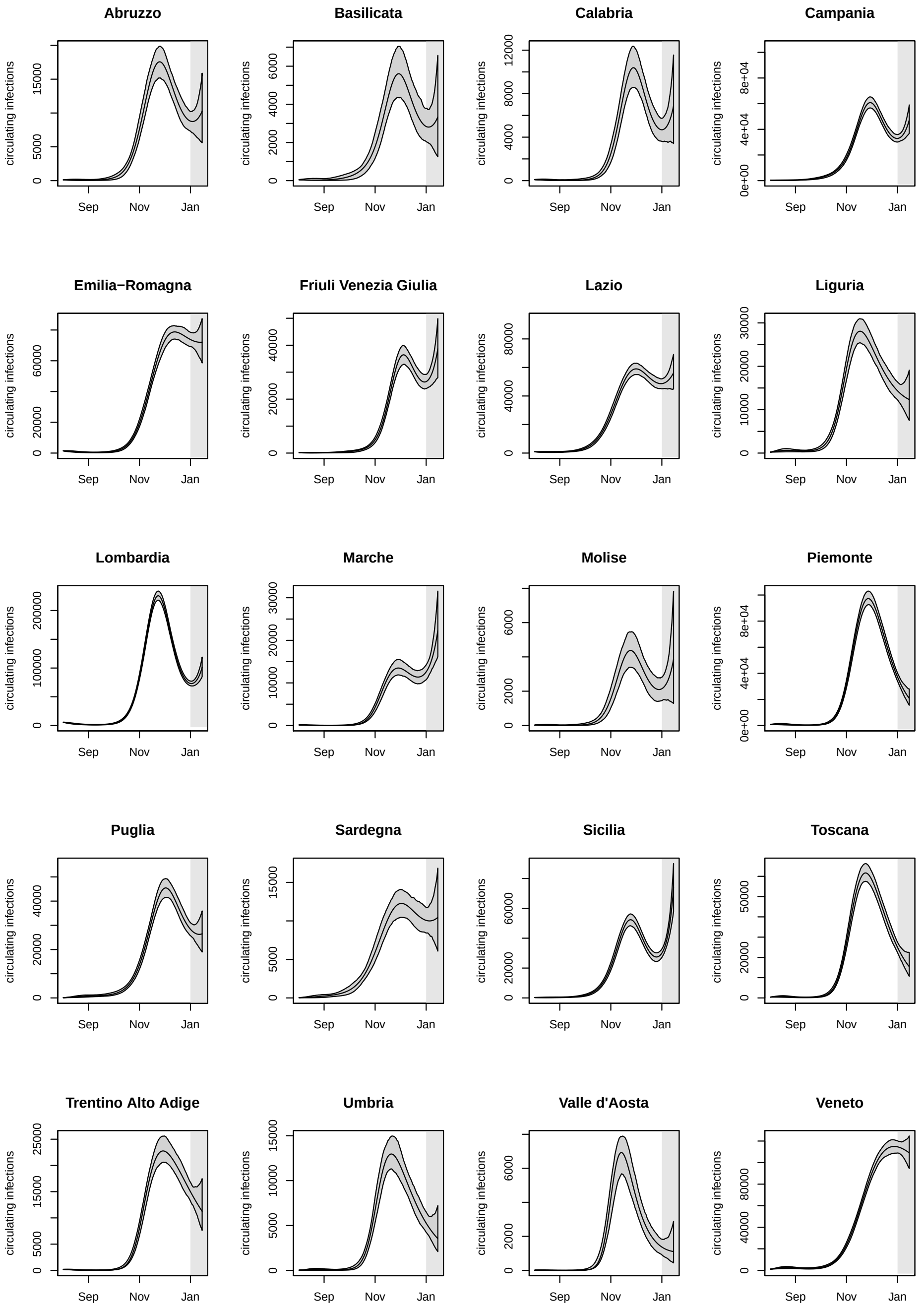

Figure S4. Estimated number of prevalent infections with pointwise 90% confidence bands, by region; $p = 1.15\%$, $T = 14$ days.

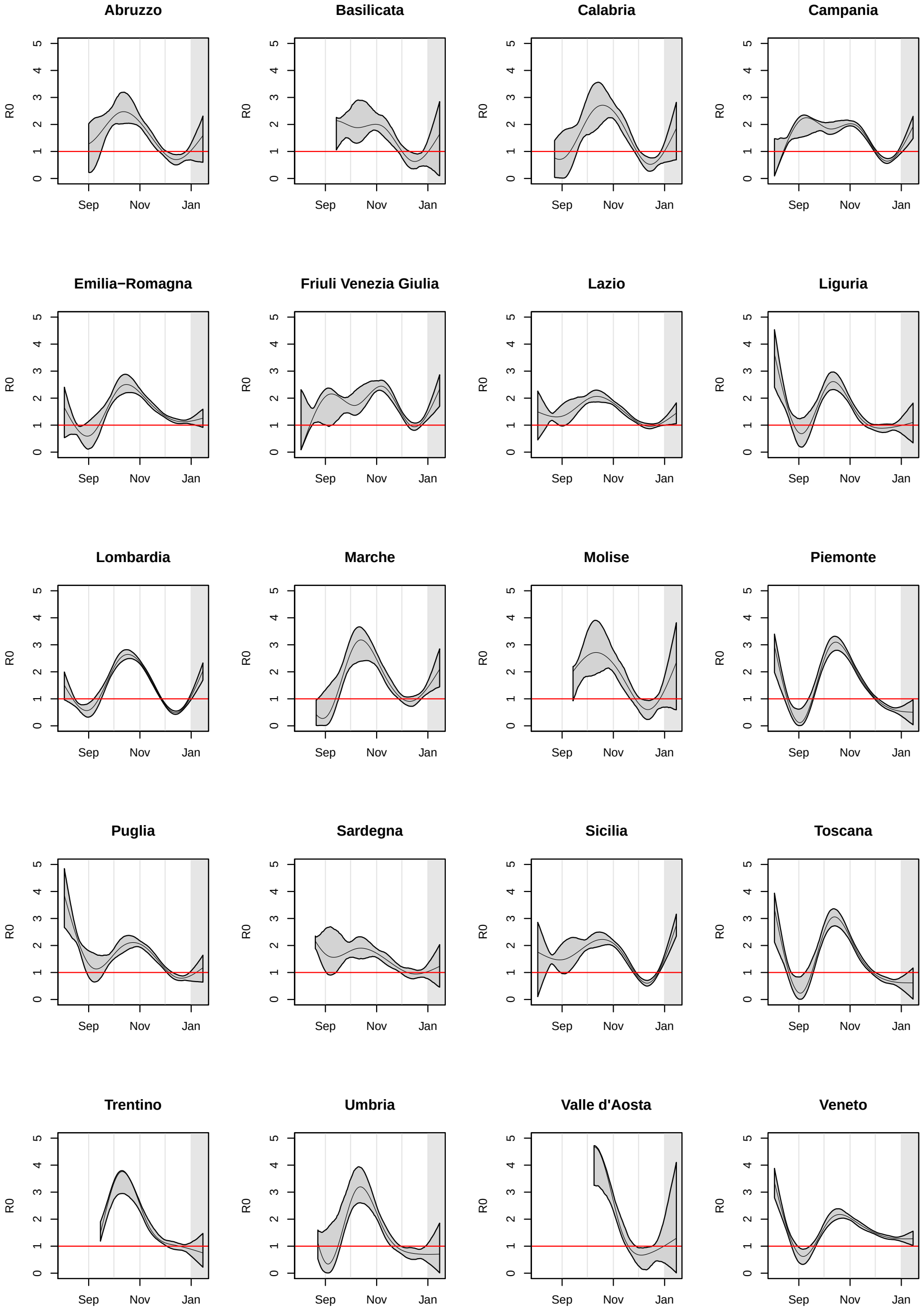

Figure S5. Estimated $R_0(t)$ with pointwise 90% confidence bands, by region; $p = 0.5\%$, $T = 14$ days.

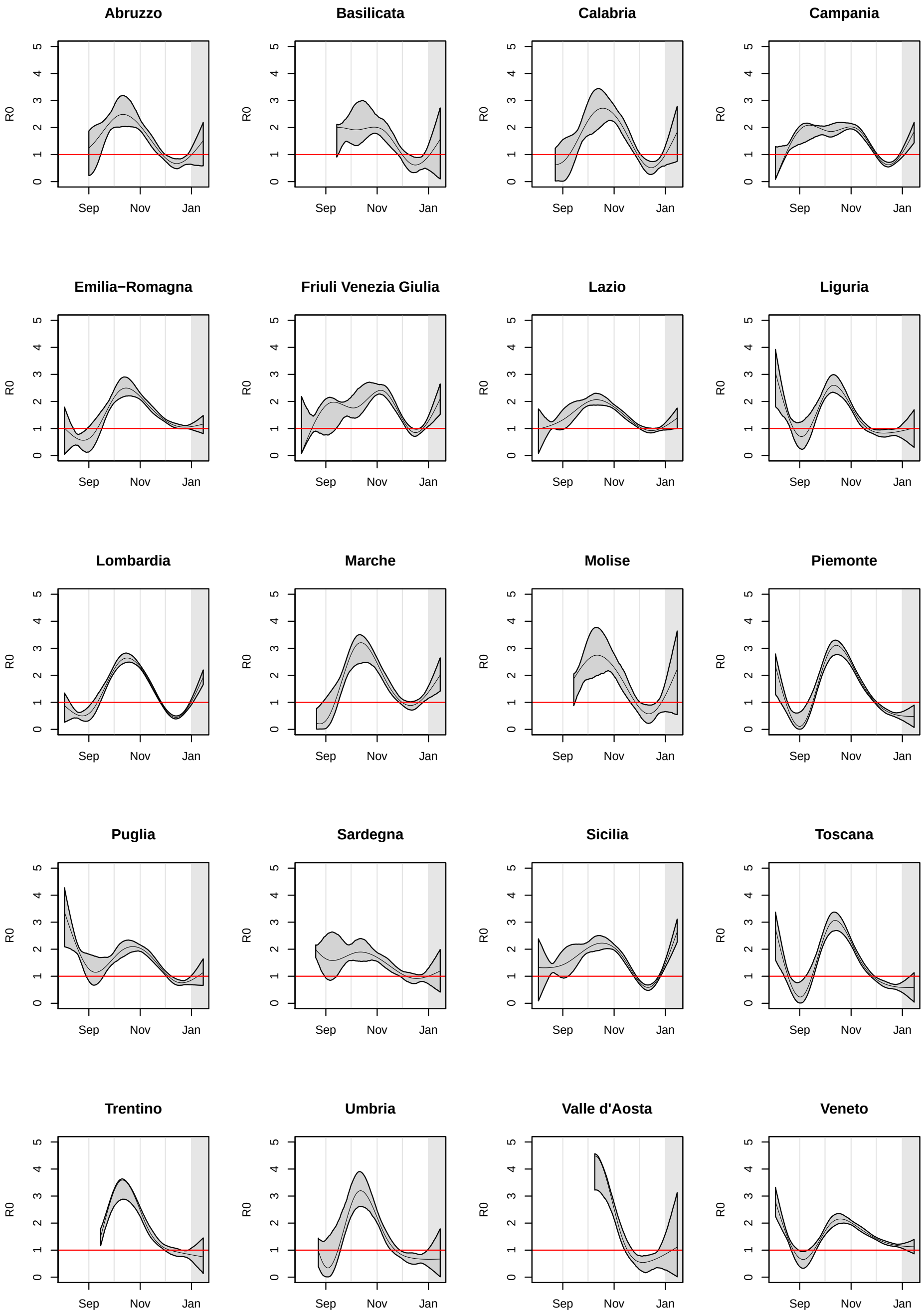

Figure S6. Estimated $R_0(t)$ with pointwise 90% confidence bands, by region; $p = 0.78\%$, $T = 14$ days.

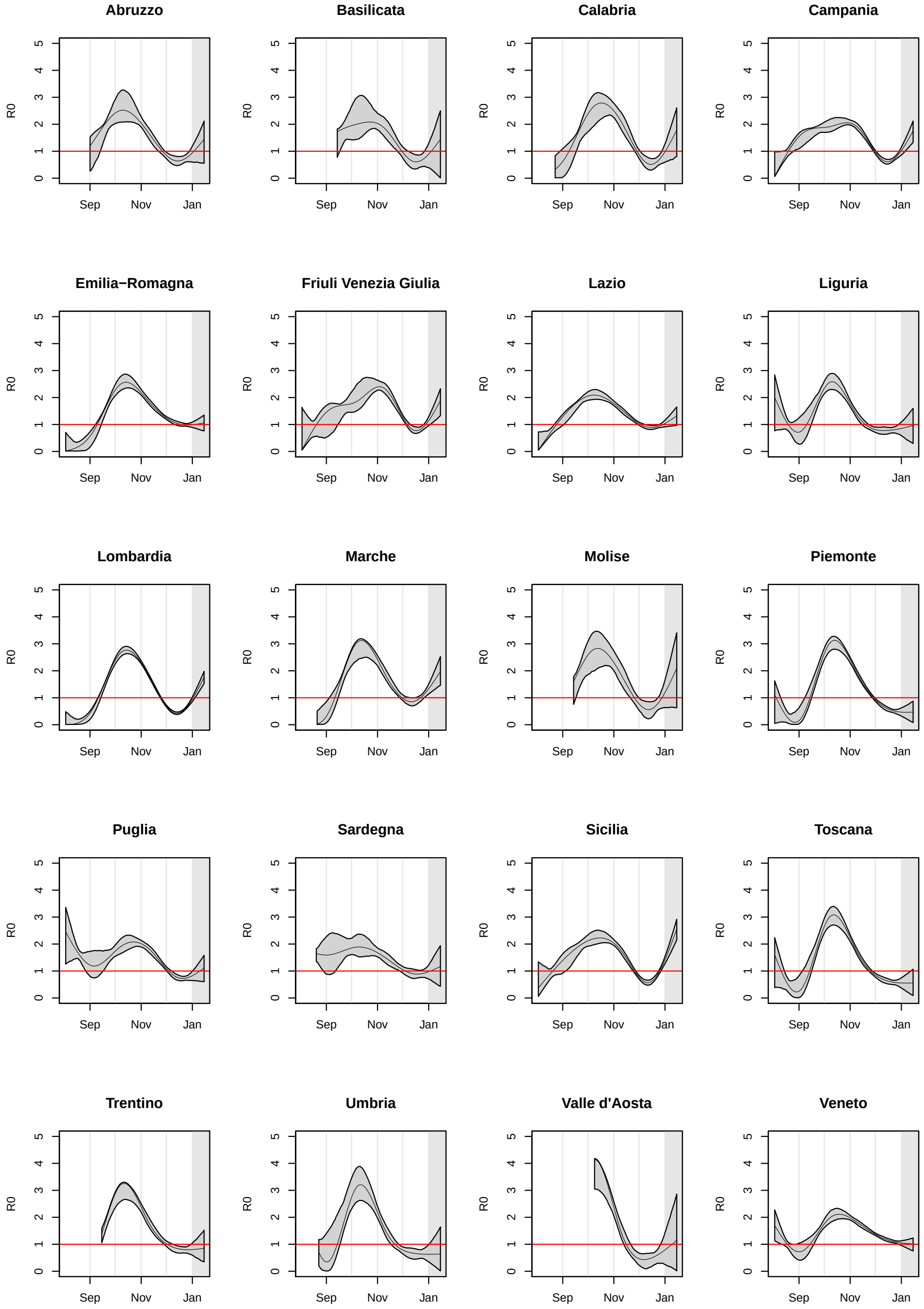

Figure S7. Estimated $R_0(t)$ with pointwise 90% confidence bands, by region; $p = 1.79\%$, $T = 14$ days.

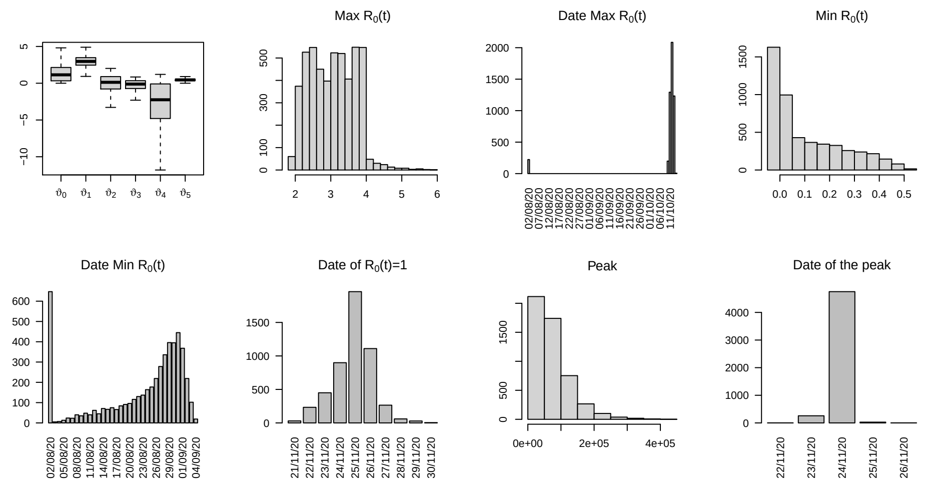

Figure S8. Monte Carlo distributions of the model outputs as the inputs vary.

TABLES

Table S1. Population size, observed number of COVID19-related deaths from August 1st 2020 to December 31st 2020, observed number of circulating infections on July 31st 2020 (i_0) and number of infections in the population notified up to July 31st 2020 (percentage of infected in population), by region.

Region	Population size	Deaths	i_0	Infections (%)
Abruzzo	1305770	741	111	3390 (0.26%)
Basilicata	556934	228	50	453 (0.08%)
Calabria	1924701	375	97	1267 (0.07%)
Campania	5785861	2409	395	4999 (0.09%)
Emilia-Romagna	4467118	3295	1496	29696 (0.66%)
Friuli-Venezia Giulia	1211357	1297	122	3394 (0.28%)
Lazio	5865544	2906	945	8646 (0.15%)
Liguria	1543127	1324	200	10214 (0.66%)
Lombardia	10103969	8317	6011	96213 (0.95%)
Marche	1518400	584	147	6886 (0.45%)
Molise	302265	168	27	471 (0.16%)
Trentino	1074819	984	202	7315 (0.68%)
Piemonte	4341375	3793	799	31667 (0.73%)
Puglia	4008296	1920	95	4614 (0.12%)
Sardegna	1630474	613	33	1415 (0.09%)
Sicilia	4968410	2129	275	3685 (0.07%)
Toscana	3722729	2539	387	10483 (0.28%)
Umbria	880285	544	18	1466 (0.17%)
Valle d'Aosta	125501	233	12	1208 (0.96%)
Veneto	4907704	4465	1000	20119 (0.41%)

Table S2. Description of meta-regressors and corresponding source.

Domain	Meta-regressor	Year	Source
General health	Percentage of people with at least two chronic diseases	2019	www.istat.it
Health infrastructures	Number of general practitioners (per 10'000 residents)	2018	
	Number of pediatricians (per 10'000 children)	2018	
	Number of hospitals (per 1'000 inhabitants)	2019	
	Percentage of public hospitals (over all hospitals)	2019	
Population age	Aging index [a]	2020	
	Mean age in the population	2020	
Household size	Average number of persons per household	2020	
Education	People with an academic degree (per 10'000 people aged 24-65)	2020	
	Schooling rate [b]	2020	
Kindergarten	Percentage of children 0-3 years old attending kindergartens	2017	
Public Transport	Percentage of people using public transport to go to work (%)	2020	
	Percentage of people aged 0-34 using public transport to go to school (%)	2020	
Economic status	Poverty index [c]	2019	
	Employment rate [d]	2018	
Tourism	Tourism rate [e]	2018	
Energy consumption	Electric energy consumption of industries and manifactures (GWH)	2018	
Density	Percentage of people living in high urbanization areas	2011	
Temperature	Average temperature (period October-December)	2020	www.Ilmeteo.it

a. Number of over 65 per 100 individuals younger than 15. b. Percentage of individuals aged 20-24 who have at least a high school diploma. c. People living in households below the poverty threshold (%). d. Employed persons in the class of age 15-64 (%, annual average). e. Days of presence of tourists in a year per inhabitant.

Table S3. Prevalence of infections (number of infections over 1,000 inhabitants) with 90% confidence intervals on September 1st, October 1st, November 1st, December 1st, and January 1st, by region; $p = 1.15\%$, $T = 14$ days.

	September 1st			October 1st			November 1st			December 1st			January 1st		
Region	prev.	90% CI		prev.	90% CI		prev.	90% CI		prev.	90% CI		prev.	90% CI	
Abruzzo	0.06	0.03	0.13	0.36	0.13	0.65	6.22	5.12	7.66	12.73	11.07	14.31	6.71	5.50	7.83
Basilicata	0.07	0.02	0.18	0.41	0.10	0.74	3.44	2.30	4.84	9.95	7.79	12.44	5.06	3.62	6.81
Calabria	0.01	0.01	0.04	0.05	0.02	0.12	1.42	0.98	1.89	5.24	4.38	6.20	2.44	1.87	2.99
Campania	0.07	0.05	0.11	0.44	0.29	0.54	3.36	3.00	3.78	10.40	9.69	11.15	5.70	5.22	6.24
Emilia R.	0.10	0.06	0.15	0.26	0.15	0.38	4.67	4.13	5.29	16.55	15.38	17.65	16.46	15.46	17.55
Friuli V. G.	0.09	0.06	0.19	0.47	0.17	0.71	4.45	3.54	5.38	29.64	26.61	32.43	22.03	19.88	24.23
Lazio	0.13	0.09	0.19	0.62	0.46	0.77	4.89	4.40	5.45	10.06	9.38	10.74	8.31	7.69	8.89
Liguria	0.34	0.19	0.50	0.84	0.53	1.20	13.29	11.48	14.84	15.63	14.17	17.24	9.16	7.77	10.48
Lombardia	0.14	0.10	0.18	0.39	0.29	0.47	9.05	8.58	9.60	19.67	18.96	20.43	7.22	6.82	7.62
Marche	0.01	0.01	0.04	0.05	0.04	0.14	3.08	2.39	3.69	8.81	7.75	10.10	8.48	7.24	9.59
Molise	0.03	0.01	0.12	0.19	0.05	0.48	5.63	3.76	7.86	13.31	10.35	16.88	7.20	4.89	9.50
Piemonte	0.02	0.02	0.07	0.11	0.10	0.26	8.13	6.76	9.36	21.08	19.18	23.73	13.68	11.95	15.35
Puglia	0.09	0.05	0.15	0.16	0.13	0.27	8.07	7.34	8.82	21.68	20.62	22.93	8.18	7.45	8.90
Sardegna	0.20	0.11	0.30	0.43	0.30	0.61	3.65	3.16	4.12	11.34	10.35	12.30	6.98	6.34	7.67
Sicilia	0.16	0.05	0.27	0.65	0.34	0.98	3.82	3.06	4.69	7.52	6.42	8.65	6.16	5.18	7.18
Toscana	0.08	0.04	0.12	0.38	0.26	0.51	4.53	4.01	5.10	9.70	8.94	10.47	6.23	5.65	6.79
Trentino	0.10	0.05	0.15	0.19	0.13	0.31	7.91	7.06	8.80	15.43	14.37	16.47	6.44	5.74	7.14
Umbria	0.08	0.01	0.18	0.17	0.06	0.38	8.24	6.58	9.96	13.01	11.17	15.08	5.81	4.69	7.21
Valle d A.	0.02	0.01	0.08	0.17	0.15	0.68	36.45	26.60	43.92	33.00	27.02	41.14	10.68	6.89	14.50
Veneto	0.45	0.33	0.57	0.56	0.42	0.74	5.15	4.66	5.69	18.66	17.74	19.69	23.32	22.17	24.40

Table S4. Mean Absolute Percentage Error (MAPE) by region and total, for prediction horizons (h) of 7 and 14 days.

	MAPE	
Region	$h = 7$	$h = 14$
Abruzzo	4.1	4.4
Basilicata	4.2	4.1
Calabria	5.3	8.2
Campania	6.2	12.9
Emilia-Romagna	6.7	13.1
Friuli Venezia Giulia	11.6	17.5
Lazio	4.6	7.8
Liguria	11.6	20.7
Lombardia	10.3	15.8
Marche	5.2	7.9
Molise	5.3	6.2
Trentino	6.6	8.4
Piemonte	4.3	7.5
Puglia	5.6	10.8
Sardegna	8.2	11.2
Sicilia	5.7	10.4
Toscana	7.3	12.7
Umbria	5.0	7.0
Valle d'Aosta	22.6	26.0
Veneto	3.3	5.3
Total	7.2	10.9

Table S5. Date of schools re-opening, by region (`studenti.it`).

Region	School beginning
Abruzzo	2020-09-24
Basilicata	2020-09-24
Calabria	2020-09-24
Campania	2020-09-24
Emilia-Romagna	2020-09-14
Friuli Venezia Giulia	2020-09-16
Lazio	2020-09-14
Liguria	2020-09-14
Lombardia	2020-09-14
Marche	2020-09-14
Molise	2020-09-14
Trentino	2020-09-07
Piemonte	2020-09-14
Puglia	2020-09-24
Sardegna	2020-09-22
Sicilia	2020-09-14
Toscana	2020-09-14
Umbria	2020-09-14
Valle d'Aosta	2020-09-14
Veneto	2020-09-14

Table S6. Posterior mean and 90% Credible Interval of the variation in the average value of $R_0(t) \times 100$ from October to December, associated to a change of one interquartile range (IQR) of each meta-regressor, posterior probability of a positive or a negative meta-regression coefficient (ζ_1). $p = 1.15\%$, $T = 14$ days.

Meta-regressor	Posterior variation	90% CrI		$\Pr(\zeta_1 > 0)$	$\Pr(\zeta_1 \leq 0)$	IQR
Two chronic diseases	-7.82	-14.59	-1.03	0.031	0.969	3.27
General practitioners	-10.44	-18.49	-2.20	0.020	0.980	1.14
Pediatricians	-7.55	-17.52	2.72	0.108	0.892	1.72
Hospitals	-3.17	-9.08	2.93	0.184	0.816	0.05
Public hospitals	-5.20	-12.90	2.39	0.128	0.872	0.16
Aging index	-0.83	-8.78	7.15	0.432	0.568	41.47
Mean age	1.46	-6.20	9.19	0.630	0.370	1.90
Household size	-2.45	-9.99	5.18	0.291	0.709	0.20
Academic degree	1.59	-3.95	7.16	0.687	0.313	0.15
Schooling index	5.53	-0.65	11.45	0.932	0.068	3.72
Children in kindergartens	11.98	5.04	19.04	0.996	0.004	10.52
Public transport (workers)	-3.18	-7.96	1.60	0.131	0.869	6.55
Public transport (students)	5.76	-0.84	12.38	0.929	0.071	6.75
Poverty index	-9.45	-15.99	-2.82	0.011	0.989	10.32
Employment rate	12.69	6.01	19.51	0.998	0.002	14.65
Tourism rate	7.26	3.36	11.19	0.998	0.002	11.02
Energy consumption	-0.35	-4.28	3.53	0.440	0.560	5088.9
Urbanization	5.84	-3.13	14.64	0.862	0.138	24.25
Average Temperature	-9.79	-14.14	-5.30	0.001	0.999	3.52

Table S7. Coefficients of variation of the model outputs as the inputs vary from Monte Carlo simulations.

Spline coefficients						$R_0(t)$					Infections	
ϑ_0	ϑ_2	ϑ_3	ϑ_4	ϑ_5	ϑ_6	Max	Max (date)	Min	Min (date)	$= 1$ (date)	Peak	Date of peak
0.86	0.26	14.04	2.91	1.08	0.44	0.21	0.21	1.24	0.47	0.01	0.74	0.002

APPENDIX

S1 TIME DISCRETIZATION IN THE SIRD MODEL

Compartmental models are described by a system of differential equations as in Equation 1 (main document). The underlying continuous time of the process can be discretized evaluating the size of the compartments at fixed points in time by considering constant intervals Δt. It follows that when $\Delta t = 1$ the system of differential equations in Equation 1 is replaced by this system of difference equations:

$$\begin{cases} S(t) &= S(t-1) - \beta(t)\frac{S(t-1)}{S(0)}I(t-1) \\ I(t) &= I(t-1) + \beta(t)\frac{S(t-1)}{S(0)}I(t-1) - \alpha I(t-1) - \delta I(t-1) \\ R(t) &= R(t-1) + \alpha I(t-1) \\ D(t) &= D(t-1) + \delta I(t-1) \end{cases}. \tag{S1}$$

The system of equations in (S1) results from a two-fold approximation. First, we assume that the rates of transition are constant in each time interval. It follows that, denoting by λ the rate of occurrence of a given event, i.e. a transition from a compartment to another one, and by τ the time at which the event occurs, the size of each compartment depends on the probability $\Pr\big(\tau \in [t, t+\Delta t)\big)$. In particular, assuming that the waiting times are exponentially distributed, $\Pr\big(\tau \in [t, t+\Delta t)\big) = 1 - \exp(-\lambda \Delta t)$. When $\Delta t = 1$, this leads to the following system of difference equations:

$$\begin{cases} S(t) &= S(t-1) - \big(1 - \exp\big(\beta(t)\frac{I(t-1)}{S(0)}\big)\big)S(t-1) \\ I(t) &= I(t-1) + \big(1 - \exp\big(\beta(t)\frac{I(t-1)}{S(0)}\big)\big)S(t-1) - \big(1 - \exp\big(-\frac{1}{T}\big)\big)I(t-1) \\ R(t) &= R(t-1) + (1-p)\big(1 - \exp\big(-\frac{1}{T}\big)\big)I(t-1) \\ D(t) &= D(t-1) + p\big(1 - \exp\big(-\frac{1}{T}\big)\big)I(t-1) \end{cases}.$$

Note that a rate of transition $\lambda = \frac{1}{T}$ corresponds to the parameter of the exponential distribution modelling the minimum between time to death and time to recovery.

Finally, a second source of approximation is introduced considering $1 - \exp(-\lambda) \approx \lambda$. This approximation applies only for small values of the transition rates.

S2 PARAMETRIC BOOTSTRAP

We performed a parametric bootstrap (Efron and Tibshirani, 1994, Ch 6.5) to compute confidence intervals around the point estimates of the coefficients of the cubic splines $\boldsymbol{\vartheta}$. We relied on a parametric approach since it is a common practice in compartmental models to assume that the daily increments of deaths are distributed according to Negative Binomial distributions. In particular, we assumed:

$$d(t) \sim NB(\lambda_d(t), \omega_d(t)) \tag{S2}$$

where $d(t) = D(t) - D(t-1)$ and $NB(\lambda, \omega)$ is a Negative Binomial distribution with mean λ and clumping parameter ω.

Starting from the estimates of the model parameters $\widehat{\boldsymbol{\vartheta}}$ obtained via calibration, we computed the size of the corresponding SIRD compartments as $\widehat{S}, \widehat{I}, \widehat{R}, \widehat{D}$ and, from the estimated time series $\widehat{D}(t)$, the daily

increments $\widehat{d}(t) = \widehat{D}(t) - \widehat{D}(t-1)$. Then, we set in (S2):

$$\widehat{\lambda_d}(t) = \widehat{d}(t), \;\; \widehat{\omega_d}(t) = \widehat{d}(t-1).$$

It should be noticed that, being the variance of the Negative Binomial equal to $\lambda(1+\lambda/\omega)$, the distribution (S2) accounts for over-dispersion, especially at the beginning and at the end of the study period, when the number of deaths and new infections is small (Grenfell et al., 2002).

In order to get n bootstrap replications of the model parameters we adopted a simplified version of the bootstrap procedure detailed in Baccini et al. (2021) and proposed by Chowell (2017):

- For each t, we sampled increments $d^*(t)$ from the following Negative Binomial distribution:

$$d^*(t) \sim NB(\widehat{\lambda_d}(t), \widehat{\omega_d}(t)),$$

- We derived one bootstrap replication for the cumulative sum of these sampled increments obtaining one bootstrap sample of $D^*(t)$,
- We performed a calibration procedurere assuming the bootstrap time series $D^*(t)$ as observed, thus obtaining a bootstrap estimate for $\boldsymbol{\vartheta}$.

This procedure was repeated n times to obtain n bootstrap replicates of the unknown parameters of the SIRD model and compute their percentile intervals (Efron and Tibshirani, 1994, Ch 13). We fixed $n = 500$.

S3 VALIDATION APPROACH

Separately by region, we performed a cross validation by repeating the calibration of the SIRD parameters on time series of increasing length (training sets) and predicting each time the number of COVID19-related deaths in the next h days (test sets). More specifically, we defined the first training set as the 60 days time series following the first death. Then, we built the second training set increasing the first by 7 days, the third increasing the second of 7 days and so on. For each training set we performed a calibration on the observed COVID19-related deaths to estimate the SIRD parameters, and we used the estimated parameters to predict the number of deaths in the next h days. We set the prediction horizon h to 7 and to 14 days.

Let i be the label of the training set ($i = 1, 2, ...I$) and m_i its length. We computed the Mean Absolute Percentage Error (MAPE) (Ferrari et al., 2021) for each region:

$$MAPE(h) = \frac{100}{hI} \sum_{i=1}^{I} \sum_{t=m_i+1}^{m_i+h} \frac{|D(t, \hat{\boldsymbol{\vartheta}}^i) - D^{obs}(t)|}{D^{obs}(t)},$$

where $\hat{\boldsymbol{\vartheta}}^i$ is the estimated parameter vector obtained with calibration interval $1, ..., m_i$. The number of elements of $\hat{\boldsymbol{\vartheta}}^i$ depends on m_i, since for each training set we defined a cubic regression spline for $R_0(t)$ with a number of equi-spaced knots proportionally reduced compared to the main analysis. The total MAPE was calculated by averaging the regional ones.

S4 GLOBAL SENSITIVITY ANALYSIS

Given K_X mutually independent inputs $(X_1, X_2, ..., X_{K_X})$ and a model which, given the inputs, returns K_Y outputs $(Y_1, Y_2, ..., Y_{K_Y})$, the Global Sensitivity Analysis (GSA) based on the Sobol's decomposition

of the variance (Sobol, 1993), allows computing both the first order and the total effect index for each input X_i and output Y (suppressing, for sake of simplicity, the index of the output variable).

The first order index for the output Y and the input X_i is defined as the ratio $S_i = \frac{\mathrm{Var}(\mathrm{E}(Y|X_i))}{\mathrm{Var}(Y)}$, corresponding to the fraction of the total variance attributable to the main effect of X_i. According to the notation adopted in Saltelli et al. (2008), the outer operator Var is applied to X_i.

The total effect index represents the proportion of the total variance of Y which is due to the main effect of the input X_i and all its interactions with the other inputs. Denoted as S_i^{tot}, it is defined as

$$S_i^{\mathrm{tot}} = \frac{\mathrm{E}(\mathrm{Var}(Y|\mathbf{X}_{\sim\mathbf{i}}))}{\mathrm{Var}(Y)},$$

where $\mathbf{X}_{\sim\mathbf{i}}$ denotes the vector $(X_1, X_2, ...X_{i-1}, X_{i+1}, ...X_{K_X})$. According to the notation adopted in Saltelli et al. (2008), the outer operator E is applied to $\mathbf{X}_{\sim\mathbf{i}}$. In the case of a multivariate output ($K_Y > 1$), an overall index can be obtained for each input by averaging the single-output indexes weighted by the variance of their corresponding output (Lamboni et al., 2011; Gamboa et al., 2014).

S5 BAYESIAN META-ANALYSIS

S5.1 Multivariate Bayesian meta-analysis

Let $\boldsymbol{\vartheta}_r$ be the vector of the spline coefficients for region r arising from the SIRD model, and Σ_r the associated estimate of the variance-covariance matrix resulting from the bootstrap replicates. We assumed the following Normal-Normal multivariate model:

$$\begin{aligned} \boldsymbol{\vartheta}_r &\sim N(\boldsymbol{\mu}_{\boldsymbol{\vartheta_r}}, \Sigma_r) \\ \boldsymbol{\mu}_{\boldsymbol{\vartheta_r}} &\sim N(\boldsymbol{\vartheta}_{all}, \Gamma), \quad r = 1, 2, ..., 20, \end{aligned}$$

where $\boldsymbol{\vartheta}_{all}$ and Γ are the overall meta-analytic vector of the spline coefficients and the between-region variance-covariance matrix, respectively. We used a MCMC algorithm to get a sample from the joint posterior distribution of $\boldsymbol{\vartheta}_{all}$ and Γ, through the function *mvma.bayesian* of the `altmeta` library of R software (Lin and Chu, 2021). This function assigns vague non informative priors on $\boldsymbol{\vartheta}_{all}$ and Γ (Lin and Chu, 2021). The same multivariate meta-analysis approach was also used to combine the regional estimates of the monthly average prevalence of infection from September to December (in this case, we combined vectors of 4 estimates).

S5.2 Bayesian univariate meta-analyses and meta-regressions

Let q_r and s_r^2 be the estimates of the quantity of interest and of its variance in the region r. The random effects meta-analysis model assumed:

$$\begin{aligned} q_r &\sim N(\mu_{q_r}, s_r^2) \\ \mu_{q_r} &\sim N(\zeta, \tau^2), \end{aligned}$$

where ζ is the overall meta-analytic quantity and τ^2 the between-region heterogeneity variance. Non-informative priors were assumed on the model hyperparameters: $\zeta \sim N(0, 10^3)$, $\frac{1}{\tau^2} \sim IG(10^2, 10^2)$. The percentage of variability due to the between region heterogeneity was expressed in terms of I^2 index

($I^2 = 100 \times \frac{\tau^2}{\tau^2+\hat{\sigma}^2}$, where $\hat{\sigma}^2$ is the harmonic mean of the variances s_r^2). The previous model can be extended to include the meta-regressor x_r:

$$q_r \sim N(\mu_{q_r}, s_r^2)$$
$$\mu_{q_r} \sim N(\zeta_0 + \zeta_1 x_r, \tau_{res}^2),$$

where x_r, ζ_0 is the overall meta-analytic estimate of the quantity of interest (in our analysis, the average $R_0(t)$ from October 1st to December 31st) when $x_r = 0$, ζ_1 is the meta-regression coefficient, and τ_{res}^2 is the residual heterogeneity variance (the between-region heterogeneity which is not explained by x_r). Non-informative priors were assumed on the model hyperparameters: $\zeta_0 \sim N(0, 10^3)$, $\zeta_1 \sim N(0, 10^3)$), $\tau_{res}^2 \sim IG(10^2, 10^2)$.

We used the software WinBUGS and the interface library R2WinBUGS of R software (Sturtz et al., 2005) to get a sample from the joint posterior distribution of the meta-analysis and meta-regression hyperparameters via MCMC algorithms (3 chains of 7'000 iterations, after a burning of 3000 iterations, and a thinning of 5).

REFERENCES

Baccini, M., Cereda, G., and Viscardi, C. (2021). The first wave of the sars-cov-2 epidemic in tuscany (italy): A si2r2d compartmental model with uncertainty evaluation. *PLOS ONE* 16, e0250029–

Chowell, G. (2017). Fitting dynamic models to epidemic outbreaks with quantified uncertainty: A primer for parameter uncertainty, identifiability, and forecasts. *Infectious Disease Modelling* 2, 379–398. doi:https://doi.org/10.1016/j.idm.2017.08.001

Efron, B. and Tibshirani, R. (1994). *An Introduction to the Bootstrap* (Boca Raton: CRC press)

Ferrari L., Gerardi G., Manzi G., Micheletti A., Nicolussi F., Biganzoli E., et al. (2021) Modeling Provincial Covid-19 Epidemic Data Using an Adjusted Time-Dependent SIRD Model. *International Journal of Environmental Research and Public Health* 18, 6563.

Gamboa, F., Janon, A., Klein, T., and Lagnoux, A. (2014). Sensitivity analysis for multidimensional and functional outputs. *Electron. J. Statist.* 8, 575–603. doi:10.1214/14-EJS895

Grenfell, B. T., Bjørnstad, O. N., and Finkenstädt, B. F. (2002). Dynamics of measles epidemics: Scaling noise, determinism, and predictability with the TSIR model. *Ecological Monographs* 72, 185–202

Lamboni, M., Monod, H., and Makowski, D. (2011). Multivariate sensitivity analysis to measure global contribution of input factors in dynamic models. *Reliability Engineering and System Safety* 96, 450–459

Lin, L. and Chu, H. (2021). *altmeta: Alternative Meta-Analysis Methods*. https://CRAN.R-project.org/package=altmeta

[Dataset] Protezione Civile. Repository of Covid-19 outbreak data for Italy. https://github.com/pcm-dpc/COVID-19

Saltelli, A., Ratto, M., Andres, T., Campolongo, F., Cariboni, J., Gatelli, D., et al. (2008). *Global Sensitivity Analysis: The Primer* (Wiley)

Sobol, I. M. (1993). Sensitivity estimates for nonlinear mathematical models. *Mathematical Modeling and Computational Experiment* 1, 407–414.

Sturtz, S., Ligges, U., and Gelman, A. (2005). R2WinBUGS: A Package for Running WinBUGS from R. *Journal of Statistical Software* 12, 1–16
